# The emerging role of neutrophil extracellular traps in autoimmune and autoinflammatory diseases

**DOI:** 10.1002/mco2.70101

**Published:** 2025-03-06

**Authors:** Liuting Zeng, Wang Xiang, Wei Xiao, Yang Wu, Lingyun Sun

**Affiliations:** ^1^ Department of Rheumatology and Immunology Nanjing Drum Tower Hospital Chinese Academy of Medical Sciences & Peking Union Medical College Nanjing China; ^2^ Department of Rheumatology Changde Hospital, Xiangya School of Medicine, Central South University (The first people's hospital of Changde city) Changde City China; ^3^ Department of Rheumatology, Peking Union Medical College Hospital Chinese Academy of Medical Sciences & Peking Union Medical College Beijing China; ^4^ Department of Rheumatology and Immunology The First Affiliated Hospital of Anhui Medical University Anhui China

**Keywords:** autoimmune kidney disease, autoimmune skin disease, autoinflammatory and autoimmune diseases, inflammatory arthritis, neutrophil extracellular traps, systemic vasculitis

## Abstract

Neutrophil extracellular traps (NETs) are unique fibrous structures released by neutrophils in response to various pathogens, exhibiting both anti‐inflammatory and proinflammatory effects. In autoimmune conditions, NETs serve as crucial self‐antigens triggering inflammatory cascades by activating the inflammasome and complement systems, disrupting self‐tolerance mechanisms and accelerating autoimmune responses. Furthermore, NETs play a pivotal role in modulating immune cell activation, affecting adaptive immune responses. This review outlines the intricate relationship between NETs and various diseases, including inflammatory arthritis, systemic autoimmune diseases, Behçet's disease, systemic lupus erythematosus, autoimmune kidney diseases, autoimmune skin conditions, systemic sclerosis, systemic vasculitis, and gouty arthritis. It highlights the potential of targeting NETs as a therapeutic strategy in autoimmune diseases. By examining the dynamic balance between NET formation and clearance in autoimmune conditions, this review offers critical insights and a theoretical foundation for future research on NET‐related mechanisms. Advances in systems biology, flow cytometry, and single‐cell multiomics sequencing have provided valuable tools for exploring the molecular mechanisms of neutrophils and NETs. These advancements have renewed focus on the role of neutrophils and NETs in autoimmune diseases, offering promising avenues for further investigation into their clinical implications.

## Graphical abstract

Neutrophil extracellular traps (NETs) released by neutrophils in response to pathogens exhibit dual anti‐inflammatory and proinflammatory effects. While effective in pathogen capture and inflammation resolution, imbalanced NET formation can lead to tissue damage and exacerbate inflammatory responses in autoimmune diseases. NETs also serve as self‐antigens, triggering immune activation and influencing adaptive responses in diseases like rheumatoid arthritis and systemic lupus erythematosus, underscoring their critical role in autoimmune pathogenesis.

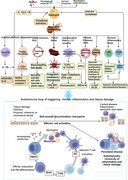

## 1 INTRODUCTION

Neutrophils are one of the most important innate immune cells in the human body. When infection and inflammation occur, they are recruited to the disease site for the first time under the action of chemokines, and participate in host immunity through phagocytosis, degranulation, and so on, which is the first line of immune defense.^1^, ^2^ During the infection of pathogenic microorganisms such as bacteria, fungi and viruses, neutrophils exert their innate immunity by phagocytosing and killing and secreting antibacterial substances.^3^ In recent years, studies have found that neutrophils can secrete a variety of cytokines to play an immunoregulatory role, and the immunoregulatory function of neutrophils has received more and more attention.^4^ As the most abundant immune cells in the circulatory system, neutrophils are essential in defending against a wide range of pathogens. However, to maintain immune balance, their antimicrobial responses must be tightly regulated.^5^ When activated by proinflammatory signals, neutrophils initiate different effector functions based on their surface receptor composition and endogenous proteins. For instance, after phagocytosing microbes, neutrophils produce reactive oxygen species (ROS), undergo degranulation, or release neutrophil extracellular traps (NETs).^6^ NETs are extracellular structures composed primarily of DNA, granule proteins, and nuclear proteins, which are assembled on a decondensed chromatin scaffold.^6^, ^7^ NETs can capture, immobilize, inactivate, and kill invading microorganisms, which are forms of innate responses against pathogen invasion and play an important role in host defense.^8^, ^9^ As a newly discovered anti‐infection mechanism of neutrophils, NETs can network pathogenic bacteria to limit their dissemination range and increase the concentration of local antibacterial substances to eliminate pathogenic bacteria. While NETs exert extracellular antibacterial effects, restricting the diffusion of various antibacterial granule proteins can reduce tissue damage caused by neutrophils, but at the same time, the formation and clearance of NETs can aggravate the severity of some autoimmune diseases.^10^, ^11^, ^12^, ^13^ In addition, cytokines secreted by neutrophils and the production of antineutrophil cytoplasmic antibodies (ANCAs) in the body also play an important role in the pathogenesis of autoimmune diseases.^14^

Emerging evidence highlights the critical role of NETs in various autoimmune diseases. NETs contribute to excessive systemic inflammation, mediate endothelial damage across multiple organs, and promote thrombosis. Their dysregulated release during neutrophil activation plays a central role in the initiation and progression of systemic autoimmune diseases. Furthermore, disruptions in neutrophil death processes lead to the modification of self‐antigens and their presentation to the adaptive immune system. Distinct neutrophil subpopulations, such as low‐density neutrophils (LDNs), further exacerbate disease pathology by promoting vascular damage and increasing oxidative stress. Advances in multiomics, single‐cell transcriptomics, and spatial transcriptomics are shedding light on the complexity of neutrophil biology and pathology.^15^, ^16^ These tools enable a deeper understanding of the roles played by different neutrophil subtypes in the pathogenesis of autoimmune and autoinflammatory diseases.^17^ Understanding the functions of neutrophils and NETs in dysregulated chronic inflammatory responses is crucial for developing targeted therapies. Such treatments have the potential to alleviate clinical manifestations and improve outcomes for patients suffering from these debilitating diseases.

**FIGURE 1:**
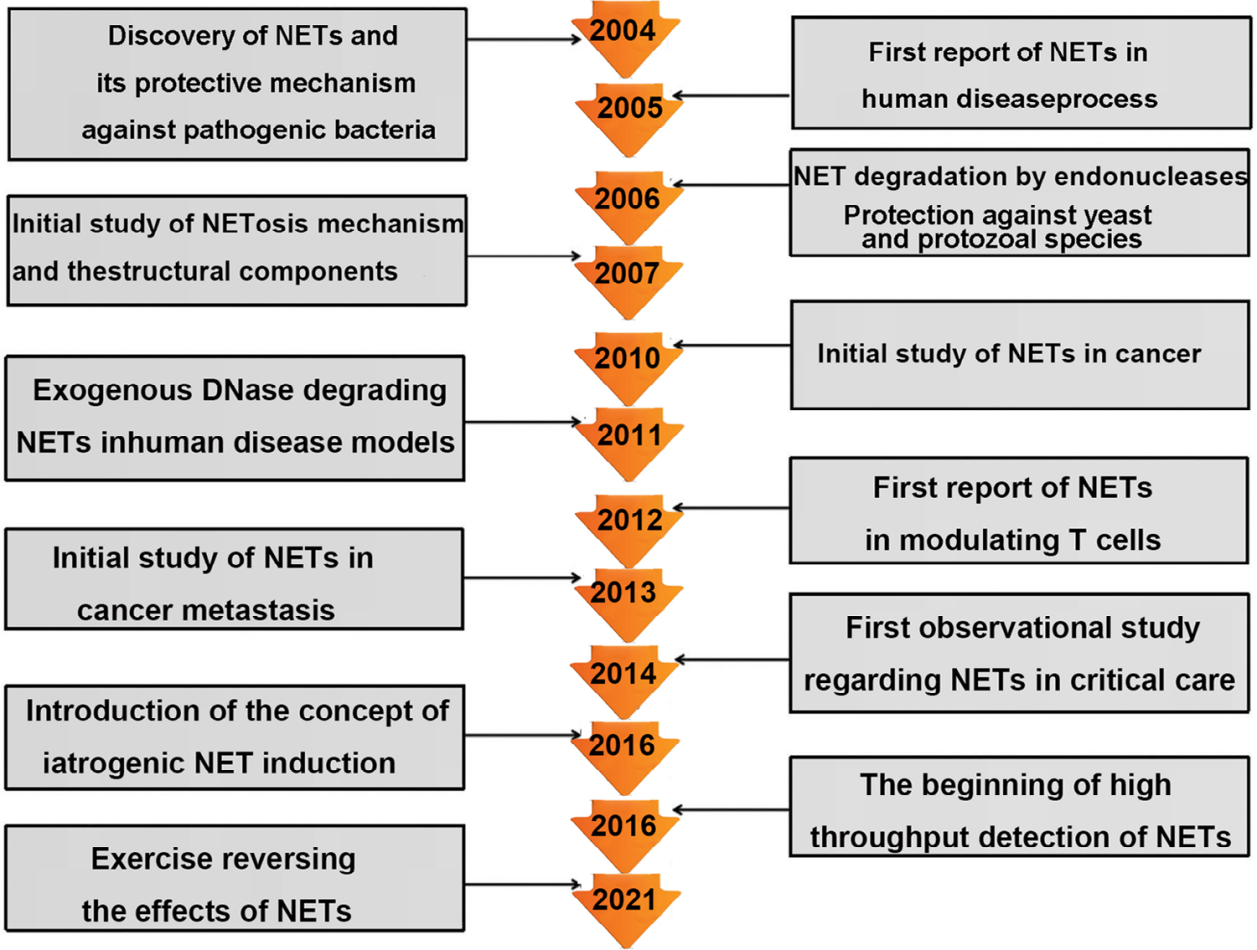
Timeline of NETs research. (Major discoveries related to NETs are illustrated, spanning their initial identification and role in pathogen elimination to their association with autoimmune and other diseases. It highlights the progression of research over time and the increasing recognition of NETs’ clinical relevance.)

This review provides a concise overview of the role of neutrophils, including NETosis, in host defense following infection and their critical contributions to both innate and adaptive immunity. It further explores the molecular mechanisms of NET formation and clearance, as well as the involvement of neutrophils in the pathological mechanisms of autoimmune and inflammatory diseases, such as inflammation, autoimmunity, thrombosis, and multiorgan tissue damage. The evidence supporting the involvement of neutrophils and NETs in the progression of various systemic autoimmune diseases is discussed, along with strategies to regulate neutrophil functions and NET formation or clearance for therapeutic purposes. Additionally, we highlight current targeted therapies aimed at NETs, offering insights into the development of NET‐focused therapeutic strategies for clinical management of autoimmune diseases. The timeline of NETs research was shown in Figure 1.

## 2 THE ROLE OF NEUTROPHILS AND NETs IN AUTOIMMUNE AND AUTOINFLAMMATORY DISEASES

**FIGURE 2:**
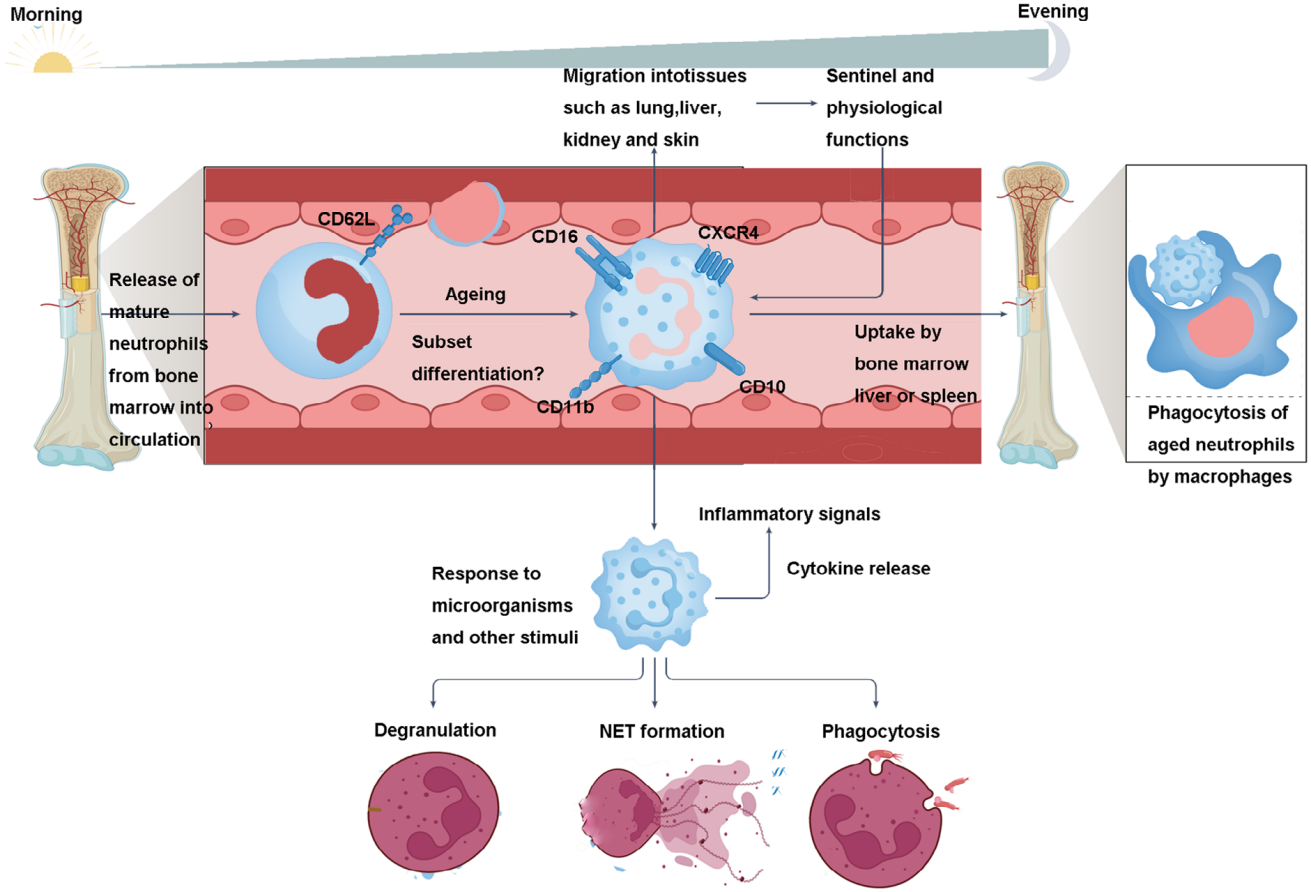
The immune function of neutrophils in autoinflammatory and autoimmune inflammation. (Mature neutrophils are released from the bone marrow into circulation, where their biology and migration to tissues are regulated by circadian rhythms but can change during inflammation. Neutrophils have a short lifespan (∼1 day), with aging marked by increased expression of surface markers like CD16, CD10, CD11b, and CXCR4. They enter tissues such as the lungs, liver, kidneys, and skin, performing functions like microbe surveillance, angiogenesis, coagulation, and tissue repair. At their lifecycle's end, neutrophils are degraded in the bone marrow, liver, or spleen. In response to inflammation, they migrate to injury sites and combat microbes via phagocytosis, degranulation, or NET formation. Created by BioRender.)

White blood cells play a crucial role in the production and development of inflammation. Among them, neutrophils can secrete a variety of inflammatory factors (peroxidase and oxygen free radicals, etc.) and a large number of inflammatory mediators, which play an important role in autoimmune diseases.^18^ Neutrophils are innate immune cells, and their main features are multilobular nuclei and contain a variety of granzymes. Its antibacterial mechanisms include ROS reaction, phagocytosis, and degranulation to release antibacterial substances.^19^ It also has MHC‐restricted and costimulatory molecules, and can act as antigen‐presenting cells to present pathogenic signals to T cells and B cells.^20^ However, the life cycle of neutrophils is very short, only 1 day. Inflammatory factors can cause damage to their own organs and tissues, and at the same time, inflammatory factors can also promote the increase of neutrophil count and platelet count.^20^, ^21^ Studies have shown that in autoimmune diseases, neutrophils secrete a variety of inflammatory factors and inflammatory mediators to activate the inflammatory response, while lymphocytes regulate the inflammatory response. Compared with single neutrophil and lymphocyte count, peripheral blood neutrophil to lymphocyte ratio (NLR) integrates the reaction process of two kinds of cells and can better reflect the autoinflammatory state.^22^, ^23^, ^24^ The higher the NLR, the greater the probability that the stable state of the corresponding inflammatory response in the body will be destroyed without the influence of other types of leukocytes in peripheral blood. Therefore, the ratio of each cell in the blood routine can be used to predict the inflammatory response process, thereby judging the disease activity^25^ (Figure 2).

**FIGURE 3:**
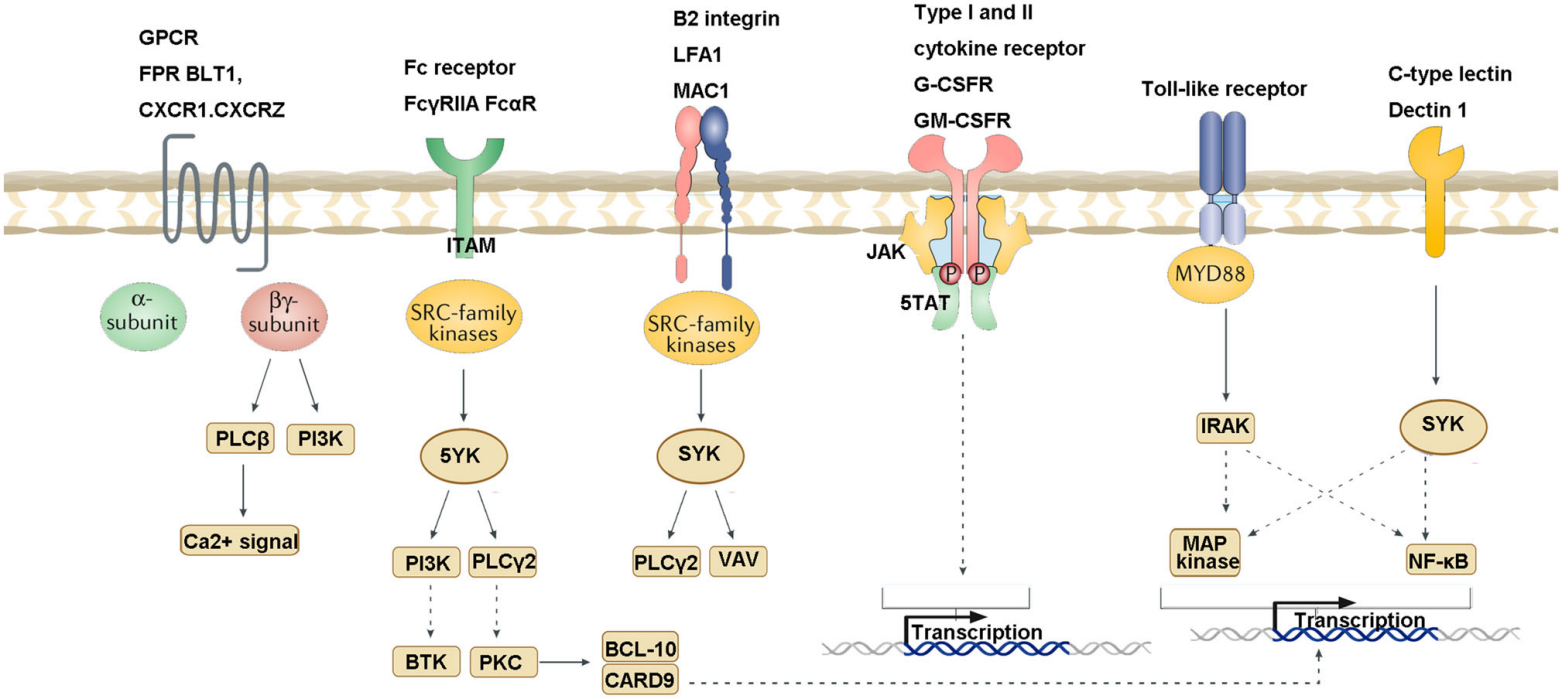
Neutrophil signaling activates surface receptors. (Neutrophils possess diverse surface receptors that regulate their functions. Key receptors include GPCRs, such as BLT1 (LTB4 receptor), C5aR1/2, CXCR2/4, and FPR1/2, which mediate chemotaxis. Toll‐like receptors detect pathogen‐associated patterns, while Fcγ receptors interact with IgG to regulate immune responses. Tyrosine kinases, including c‐MET, Src, and Syk, modulate signaling networks. Neutrophils detect microbial products and inflammatory signals, activating pathways like NADPH oxidase to produce ROS, which drives chromatin decondensation and NETosis via calcium signaling and serine proteases like NE and MPO. Created by BioRender.)

Neutrophils are the most abundant white blood cells in the human body, with a proportion of 50–70%. Mature neutrophils have typical polymorphic nuclei. It has long been regarded as a short‐lived effector cell of the innate immune system with a major role in fighting extracellular pathogens and acute inflammation.^26^, ^27^ After being recruited to the lesion site, activated neutrophils function efficiently through phagocytosis, degranulation, release of ROS, and formation of NETs.^28^ Recent studies have shown that neutrophils are not only effector cells of innate immunity, but also an important regulator of adaptive immunity.^29^, ^30^ It plays a key role in the pathogenesis of most diseases, including infections caused by intracellular pathogens, autoimmune diseases, chronic inflammation, and cancer. Taking systemic lupus erythematosus (SLE) as an example, in autoimmune diseases, current studies have reported abnormal neutrophil function in patients.^31^ Compared with serum from healthy donors, serum from SLE patients can lead to increased neutrophil aggregation and interfere with phagocytosis and lysosomal enzyme release by normal neutrophils in vitro.^32^ Impaired phagocytic capacity of neutrophils and abnormal phagocytic clearance of neutrophil apoptosis‐producing substances that have been demonstrated in SLE disease have been suggested to play a role in the pathogenesis of SLE.^33^, ^34^ In addition, studies have shown that neutrophils in SLE are less sensitive to cytokines, including interleukin (IL)‐8, and their telomeres are shortened prematurely, suggesting enhanced aging.^35^, ^36^ In recent years, studies have found that neutrophils in SLE patients are abnormal in phenotype and function, and it is speculated that they play an important role in the occurrence and development of the disease.^37^, ^38^, ^39^ A clinical study pointed out that SLE patients were accompanied by neutropenia.^40^ Our previous study also found that the proportion of neutrophils in SLE decreased. In fact, a marked reduction in the number of neutrophils in the blood can lead to severe immunodeficiency in humans. Given the special status of neutrophils in the body's immune system, the reasons and mechanisms for these abnormal phenomena are still unclear.^41^ In autoimmune diseases, immune complexes (ICs) in the blood may accumulate in capillaries and can directly activate cells with FcR, and then act as direct actors of the immune response. Neutrophils may be candidates for this role. They are highly abundant in human blood (up to 70% of leukocytes) and express a large number of activated FcRs with little or no inhibitory FcRs. Furthermore, it has been reported that circulating ICs formed immediately after antigen challenge can be captured by neutrophils through their FcRs. Likewise, neutrophils from transgenic mice expressing the human IgG receptors FcyRIIA and FcyRIIIB bind and internalize IV‐injected ICs via either receptor.^18^ These experiments confirmed that naive neutrophils were able to bind and internalize ICs in the blood; thus, neutrophils were able to initiate inflammatory responses under the influence of ICs (Figure 3).

### 2.1 Neutrophil activation and its role in innate immunity in autoimmune diseases

When infection occurs, neutrophils are rapidly recruited from the peripheral circulation to the infection site under the action of various chemokines (such as IL‐8, IL‐1β, tumor necrosis factor [TNF], endotoxin, prostaglandins, and leukotrienes [LTs]) to exert their innate immunity.^42^ Neutrophils contain a variety of granules, including azurophilic granules (primary granules), specific granules (secondary granules), gelatinase granules, and secretory vesicles. The secretory vesicle membrane contains a variety of membrane proteins related to neutrophil activation, such as CD11b/CD18 (β2 integrin), complement receptor 1 (CR1), CD14, CD16, formyl receptor, and so on.^43^ The binding of CD62L molecules on the surface of neutrophils to P‐selectin on the surface of activated endothelial cells (ECs) activates VAMP‐2‐rich secretory vesicles to fuse with the cell membrane. This makes neutrophil activation membrane proteins upregulated, releasing neutrophil cytoplasmic proteins, further chemotactic inflammatory cells, and promoting the process of inflammatory response.^44^, ^45^ Since exocytosis of secretory vesicles at this critical stage of inflammation does not lead to the release of proteases, tissue damage can be effectively avoided. The mechanism of neutrophil activation may be related to the upregulation of intercellular adhesion molecule expression on EC surface, the increase of TNF production, the increase of platelet‐activated endothelin‐1 secretion, hyperlipidemia, and cytokines IL‐1, IL‐6, and IL‐8.^46^ In addition, complement C5a, lipopolysaccharide (LPS), PAF, f‐MLP, and so on can also activate neutrophils. Activated neutrophils engulf pathogenic microorganisms at the infection site to form phagosomes, which fuse with azurophilic granules to degranulate the latter and allow enzymes to be injected into phagosomes.^47^ Meanwhile, activated neutrophils trigger NADPH oxidase (NOX) on the cell membrane, causing a burst of neutrophil respiration and producing a large amount of ROS metabolites. Pathogenic microorganisms are killed and degraded under the combined action of enzymes and ROS.^48^ The production and release of proteases and ROS can also cause severe tissue damage.^49^

### 2.2 Neutrophil immune regulation function

**FIGURE 4:**
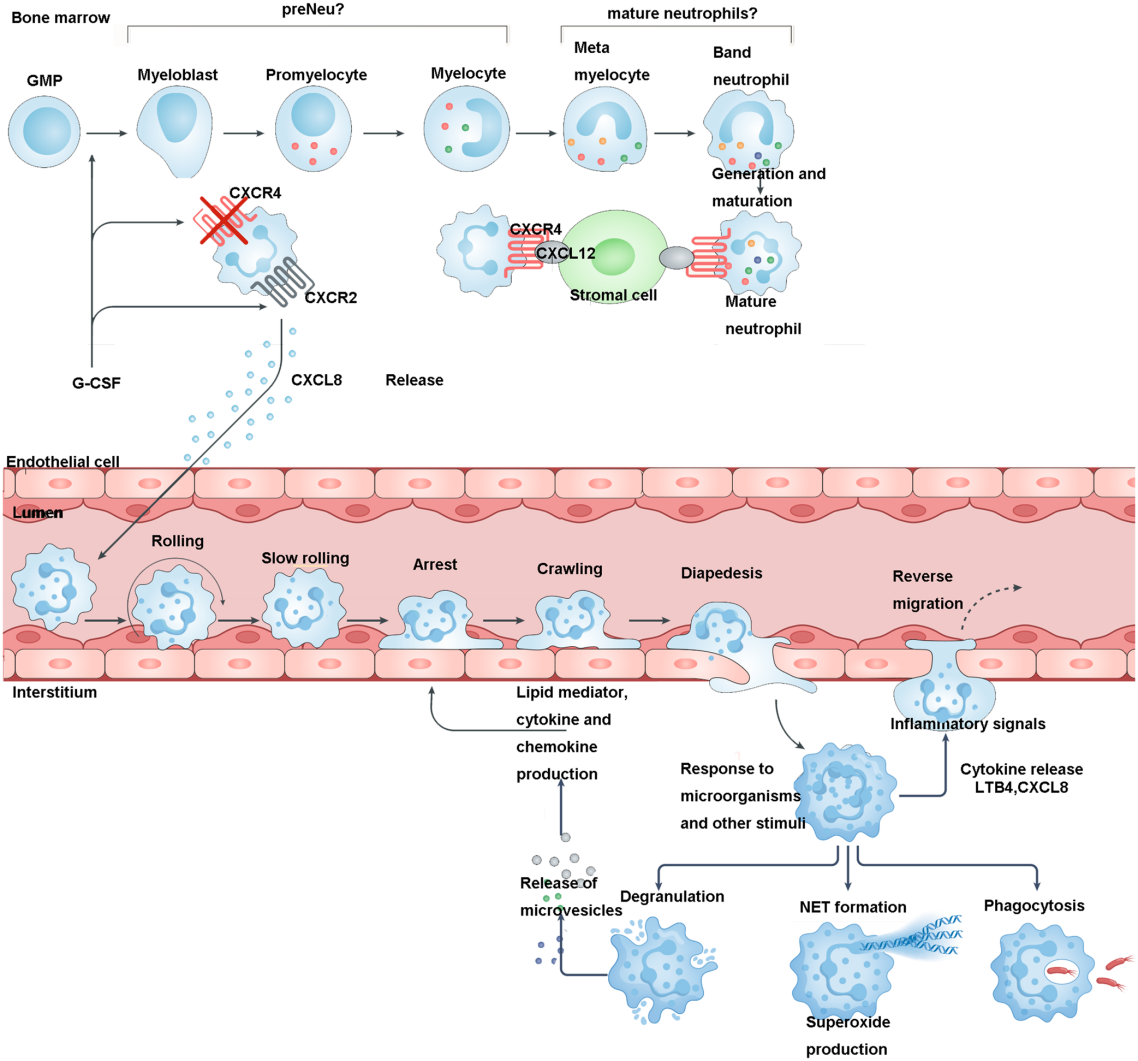
Neutrophil immune regulation function. (Inflammation or tissue damage increases G‐CSF production, mobilizing neutrophils into circulation. Selectin–receptor interactions facilitate rolling along endothelium, while chemokine gradients guide migration. Chemokine receptor activation upregulates integrins, enabling firm adhesion and transendothelial migration. At infection sites, neutrophils phagocytose pathogens, produce ROS/RNS, release antimicrobial agents via degranulation, and form NETs. Created by BioRender, with reference to Ref. ^521^.)

Neutrophils exert immunomodulatory effects by secreting a variety of cytokines. The cytokines secreted include IL‐8, TNF‐α, IL‐1α, IL‐1β, IL‐10, IL‐12, IFN‐γ, MIP‐1α, MIP‐1β, and so on.^50^ As immune cells that first arrive at the site of infection, cytokines secreted by neutrophils play an important role in the activation and recruitment of monocyte–macrophages and dendritic cells.^51^, ^52^ IL10 is a pleiotropic immunoregulatory and anti‐inflammatory cytokine, which can downregulate the expression of cytokines required for Th1 helper T cell differentiation, MHC class II molecules and macrophage activation combined stimulatory factors, and regulate the type of adaptive immune response.^53^ Neutrophils contain a small amount of IFN‐γ storage, and can synthesize and release IFN‐γ under the induction of IL‐12. Through autocrine action, IFN‐γ can stimulate the production of IL‐12, forming a positive feedback mechanism. IL‐12 and IFN‐γ costimulate Th1 cells, which is beneficial to delayed hypersensitivity reactions.^54^ In addition, IL‐12 and IFN‐γ can synergistically stimulate the proliferation and differentiation of CTL cells, thereby enhancing the subsequent cellular immune response.^55^ BAFF is a member of the TNF superfamily (also known as BLyS, THANK, TALL‐1, TNFSF13B, and zTNF4) discovered in 1999. It can specifically bind to B lymphocytes and induce their proliferation, differentiation, and secretion of immunoglobulins, and play an important role in humoral immunity.^56^ Neutrophils are one of the main cells expressing BAFF. IL‐10, IFN‐γ, and IFNα can stimulate its expression, thereby activating B lymphocytes to regulate the process of humoral immunity.^57^ BAFF exists in two forms: membrane‐bound protein and soluble ligand (hsBAFF). Studies have shown that G‐CSF in neutrophilic exudates can stimulate neutrophils to synthesize and secrete soluble B‐lymphocyte activating factor (BLyS), which is involved in autoimmune responses^58^, ^59^ (Figure 4).

## 3 NETs OVERVIEW

### 3.1 Discovery of NETs and their structural characteristics

**FIGURE 5:**
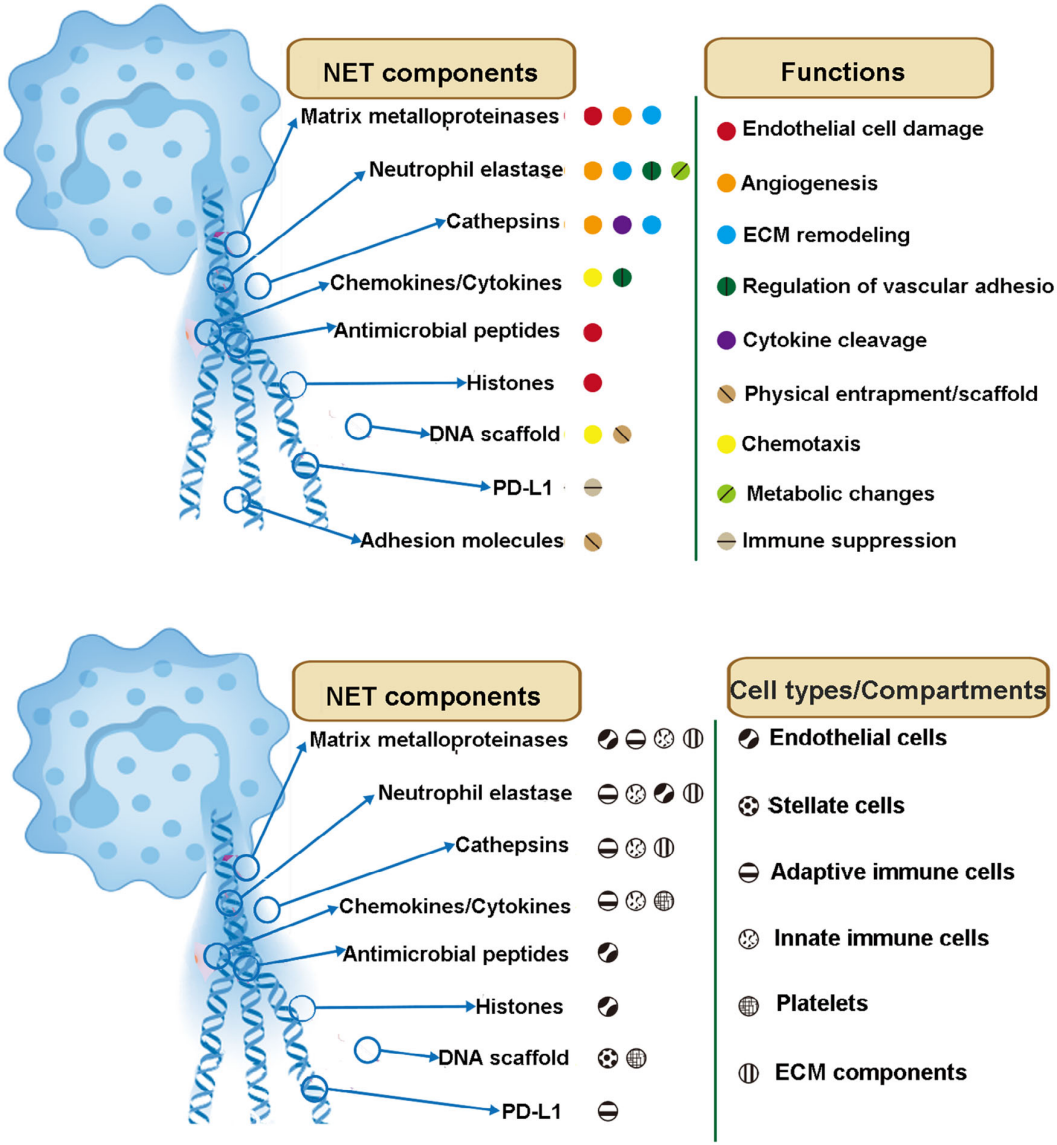
NETs and their structural characteristics. (NETs are composed of various components, with proteases being the most well‐studied. During NET formation, these proteases bind to NET–DNA, exerting cytotoxic effects on target cells and mediating proteolytic degradation of the vascular system and extracellular matrix (ECM). These processes highlight the dual roles of NETs in immune defense and tissue damage. Created by BioRender.)

In 1996, Takei et al. found that the morphological changes of neutrophils treated with phorbol ester (PMA) were completely different from apoptosis and necrosis, and eventually the cells died.^60^, ^61^ Eight years later, Brinkmann et al.^28^ found that activated neutrophils released granule proteins and chromatin to form NETs to trap bacteria. In 2007, Fuchs et al.^62^ confirmed that the death mode of neutrophils releasing NETs is different from apoptosis and necrosis, and defined it as NET‐net death (NETosis). The general process is as follows: after neutrophils are activated, the nuclear membrane is cleaved, elastase, and myeloperoxidase (MPO) in the cytoplasm enter the nucleus and act on histones to promote chromatin depolymerization. Then, it is released from the nucleus and combined with various antibacterial substances and granule proteins in the cytoplasm and released to the outside of the cell to form NETs.^63^, ^64^ High‐resolution scanning electron microscopy revealed that NETs have a unique ultrastructure. Its main components are DNA fibers with a diameter of 15–17 nm and spherical protein domains with a diameter of about 25 nm, which together constitute a three‐dimensional network structure up to 50 nm^28^ (Figure 5).

### 3.2 Initiating factors for NETs

NETs‐related substances mainly come from the nucleus, so they are highly enriched in core histones. But it also includes high levels of granular proteins (neutrophil elastase [NE], cathepsin G, and protease 3), MPO, or cytoplasmic proteins such as S100 protein.^61^ Although the NET proteome composition is fairly stable, the relative abundance of its constituent proteins and their composition may vary by stimulus. Notably, under certain stimuli, NETs may originate in mitochondria, ultimately altering NET composition and function, as these organelles lack histones. NETs are primarily produced through a cell death program known as lytic or suicidal NETosis.^28^ The process begins with the activation of surface receptors, triggering a program that completes within a few hours and performs four key tasks: penetration of the plasma membrane, disassembly of the cytoskeleton and nuclear membrane, decondensation of chromatin, and the assembly of antimicrobial proteins on chromatin scaffolds.^62^ NETosis externalizes nuclear and mitochondrial DNA. In addition to the lytic procedure, another mechanism of rapid NET release is called vital NETosis, which squeezes out the nucleus or mitochondrial DNA from living cells. The following will focus on lytic NETosis, as it is the primary mechanism of inflammatory disease.^63^

NETs are reticular structures released after neutrophil activation. Activating factors include a variety of pathogens such as Klebsiella pneumoniae, Candida albicans, Leishmania, HIV‐1, and so on,^64^ and some cytokines, chemical reagents, and bacterial components can also promote the generation of NETs, such as TNF‐α, PMA, and LPS.^65^ Studies have found that lipids can also regulate the formation of NETs. For example, changes in the lipid composition of neutrophils promote the formation of NETs.^66^ Physiological levels of 2‐chloro‐fatty acids induce human neutrophils to form reticular structures and reduce colony formation in *Escherichia coli*.^67^ Oxidized low‐density lipoprotein can significantly promote the formation of NETs in HL‐60‐derived neutrophils induced by PMA, while reduced low‐density lipoprotein does not have this function.^68^ In addition, in the presence of classical chemical inducers PMA or Staphylococcus aureus, antimicrobial peptide (AMP) LL‐37 can significantly promote the reticular formation of primary human neutrophils.^69^ Arai et al.^70^ found that low concentrations of uric acid can inhibit the formation of NETs, while high concentrations promote the production of NETs. Heme can stimulate neutrophils to release NETs in vitro and in vivo, and is involved in the pathogenesis of sickle cell disease.^71^ Antiphospholipid antibodies and antineutrophil cytoplasmic antibodies can stimulate the formation of NETs and participate in the production of thrombus in patients with antiphospholipid syndrome (APS) and the pathogenesis of antineutrophil cytoplasmic antibody‐related vasculitis, respectively.^72^ More inducers involved in disease progression by stimulating the formation of NETs remain to be discovered.

## 4 MOLECULAR MECHANISMS OF NETs FORMATION

### 4.1 ROS‐mediated MAPK signaling pathway, PI3K signaling pathway, and so on are involved in the formation of NETs

ROS play a central role as signaling mediators linking upstream regulatory pathways to the mechanisms driving NETosis. ROS is a general term for a group of highly chemically active oxygen‐containing molecules, including hypochlorous acid, peroxide, singlet oxygen, and so on, which can change their properties by interacting with almost all cellular components. The physiologically generated ROS is mainly produced by the metabolism of enzymes, such as NOX complex. Studies have shown that intracellular NOX is involved in the formation of NETs, and when NOX inhibitor (diphenylene iodide) is added, it completely blocks the release of PMA‐induced NETs after inhibiting the redox reaction.^73^ NOX‐deficient mutant mouse neutrophils fail to form NETs. Moreover, neutrophils from patients with chronic granulomatous disease (CGD) lack the membrane subunit gp91phox (i.e., NOX2) of NOX, and no NETs are produced after stimulation with PMA. When exogenous hydrogen peroxide is added, the ability to form NETs is restored, and the ability of neutrophils to form NETs in CGD patients is remodeled by gene therapy, which can restore the clearance of conidia and hyphae of Aspergillus, and promote the rapid cure of refractory invasive pulmonary aspergillosis.^74^ Other researchers have found that IL‐8 participates in the progression of glioblastoma by promoting NETs formation through PI3K/AKT/ROS axis, and that apolipoprotein E promotes infarction healing after myocardial infarction by regulating NETs formation through ROS/MAPK/MSK1 pathway.^75^, ^76^ All of these demonstrate the important role of ROS generated by NOX on the formation of NETs and thus contribute to the development of corresponding therapeutic drugs. For example, the NE inhibitor ONO‐5046 can reduce the formation of LPS‐induced NETs by inhibiting the production of ROS, thereby reducing the symptoms of inflammation.^77^ In addition, several enzymes that regulate NOX2 activity, such as protein kinase C, are also involved in reticulogenesis. However, the receptors that trigger NETs release and the molecular mechanisms by which ROS drive this process are still poorly understood.^78^

In addition to the classical ROS‐dependent pathway for NETs formation, there is also a ROS‐independent early/rapid release pathway. During NOX‐independent NETs formation, neutrophils are not lysed, but instead release NETs through nuclear membrane blebbing and subsequent inside‐out vesicular transport. Activated platelets are potent inducers of NOX‐independent network formation.^79^ In addition, C. albicans stimulation of neutrophils through Dectin‐2‐mediated NOX‐independent NETs formation contributes to the inhibition of fungal proliferation.^80^ Furthermore, NETs induced by TNF‐α,^73^ Leishmania promastigotes, and ICs^81^ are all independent of the ROS pathway. In summary, whether ROS is required for the formation of NETs depends on the type of stimuli, and the mechanism by which ROS promotes the formation of NETs remains to be further studied.

### 4.2 Activation of peptidyl arginine deiminase 4 is involved in promoting the formation of NETs

Another factor associated with NETosis is peptidyl arginine deiminase 4 (PAD4). ROS also contributes to the activation of PAD4. PAD4‐mediated citrullination can be triggered by hydrogen peroxide, and inhibition of NOX reduces citrullination, providing a link between PAD4 and ROS production.^82^ In contrast, PAD4‐mediated p67phox citrullination of p47phox promotes their dissociation from NOX to inhibit ROS production.^83^ How this negative feedback mechanism affects ROS‐mediated mechanisms and how PAD4 cooperates with other chromatin decondensation promoting factors remains unclear. PAD is a posttranslational modification enzyme that catalyzes the conversion of arginine residues in protein peptide chains to citrulline residues. There are five subtypes of PAD, among which PAD4 is involved in the formation of NETs by mediating citrullination of histone arginine residues to dedensify chromatin. However, PAD2 is involved in TNF‐α‐induced histone citrullination and is involved in the pathogenesis of arthritis, and has nothing to do with the formation of NETs.^82^ Gößwein et al.^83^ found that calcium ionophore‐induced NETs formation was significantly dependent on PAD4, because selective PAD4 inhibitors could prevent its induced chromatin depolymerization. They further found that PAD4 is dependent on calpain proteolysis for its ability to rupture the nuclear envelope and unwind chromatin.^83^ Moreover, ketamine (a broad‐spectrum PAD inhibitor) or GSK484 (a selective PAD4 inhibitor) can also prevent TNF‐α, GM‐CSF, and PMA‐induced histone H3 citrullination.^73^ Some scholars also found that after chemokine stimulation of PAD4−/− neutrophils, NETs could not be formed, and the innate immune defense ability was lost.^84^ PAD4‐deficient neutrophils were unable to release NETs under in vitro stimulation with ionomycin or PMA, but aggravated acute inflammation and increased tissue damage after myocardial infarction by producing more ROS.^85^ In addition, PAD4−/− breast cancer mice can reduce the volume and weight of primary tumors and lung metastases due to the disorder of NETs formation,^86^ and targeted inhibition of PAD4 expression can significantly improve the clinical symptoms and disease progression of Crohn's disease (CD), multiple myeloma, acute pancreatitis, and lupus model mice.^87^, ^88^, ^89^, ^90^ In summary, we know that PAD4 mediates the corresponding diseases by participating in the formation of NETs. Then, what are the related molecules that regulate the function of PAD4? Studies have shown that genetic variation in the A20 deubiquitinase domain encoded by TNFAIP3 increases susceptibility to SLE by upregulating the expression of PAD4 and the resulting protein citrullination and formation of NETs.^91^ Additionally, miR‐155 regulates the expression of PAD4 mRNA through the AU element‐rich 3′‐UTR region, therefore, targeting miR‐155 may help suppress excessive NETs production in inflammatory diseases.^92^ However, when Candida albicans^93^ and Entamoeba histolytica trophozoites^94^ were used as stimuli, the formation of NETs could also be independent of PAD4 activity. Therefore, the role of PAD4 in the formation of NETs remains to be further studied, and the mechanism of how these stimulators activate PAD4 in neutrophils and promote the formation of NETs is still unclear.

### 4.3 Gasdermin D is involved in promoting the formation of NETs

The breakdown of the cytoskeleton and the decondensation of chromatin reduce the stability of the plasma membrane. However, cell death and membrane permeabilization are mainly accelerated by inflammasome 40 and gasdermin D (GSDMD) pore assembly at the plasma membrane.^95^, ^96^ GSDMD activation in human neutrophils can trigger caspase‐11‐mediated GSDMD activation and NETosis following activation by PMA, cytosolic LPS, or virulent Gram‐negative bacteria.^97^ GSDMD is in a feedback loop with NE, NE promotes its activation, and GSDMD promotes the release of NE from azurophilic granules. Furthermore, GSDMD alters the cellular ion gradient by assembling pores in the plasma membrane, a process that may promote the activation of PAD4.^98^ NETosis also requires Caspase‐b and gasdermin Eb‐mediated pyroptosis in response to bacterial infection in zebrafish.^99^ Studies have investigated the involvement of autophagy‐related genes PI3K in autophagy 7 (ATG7) and 5 (ATG5) in NETosis, but their mechanisms in mouse or human neutrophils are unclear.^100^, ^101^, ^102^, ^103^ Neutrophils combine various elements with other processes, such as cell cycle, DNA repair, and pyroptosis, to orchestrate unique antimicrobial strategies.

### 4.4 Src/Syk, PI3K/Akt, MAPK, and ERK1/2 pathways are involved in the regulation of NETs formation

NETs are a form of death of neutrophils, and the signaling pathway proteins involved in it are the research hotspots.^104^ Zhu et al.^105^ found that 64, 97, and 141 proteins were regulated differently in neutrophils stimulated by PMA, ionomycin, and MSU, respectively. It was further found that 931, 565, and 201 phosphorylation sites were differentially regulated.^105^ Accordingly, it was found that the extracellular chromatin filaments formed by neutrophils after inhibition of TAK1, p38MAPK, and MEK pathways were short and hardly connected to each other.^73^ However, it has little effect on the release of chromatin, thereby regulating the early formation of NETs. In contrast, Syk and PI3K protein inhibitors treated neutrophils with little chromatin excretion, suggesting their involvement in the later formation of NETs. However, inhibition of PKC, Src family kinases and JNK synthesis, transcription and protein translation cannot effectively prevent the production of NETs.^73^ Specific inhibitor experiments demonstrated that JNK and PI3K were involved in the formation of NETs induced by pyocyanin. Ravindran's group^106^ found that JNK inhibitors such as SP600125 significantly interfered with LPS‐induced NETs formation, but did not affect the stimulatory effect of PMA, suggesting that the two agonists exert their effects through different mechanisms. In addition, the molecular mechanisms involved in the generation of NETs by different physiological stimulants are also different. For example, the formation of NETs stimulated by anti‐β2‐glycoprotein I (β2GPI)/β2GPI complex does not depend on p38, AKT, ERK1/2, or zinc signaling.^107^ Immobilized ICs induced NETs released from human primary neutrophils, mediated by FcγRIIIb, β2 integrin involved, and regulated by Src/Syk, PI3K/Akt, p38 MAPK, and ERK1/2 pathways.^108^ In addition, propofol can reduce PMA‐induced NETs formation by inhibiting p‐ERK and HOCl production.^109^ The Yersinia pseudotuberculosis adhesion protein invasin induces ROS production and chromatin release into the extracellular environment, mediated by β1 integrin, and is dependent on ROS and PI3K signaling.^110^ In summary, multiple cascade protein pathways are involved in the formation of NETs, but differ by stimuli.

### 4.5 Multiple signaling pathway proteins are involved in the occurrence and development of autoimmune diseases by regulating the formation of NETs

In recent years, studies have found that multiple signaling pathway proteins are involved in the occurrence and development of autoimmune diseases by regulating the formation of NETs. Studies have found that NETs are dependent on the activation of TLR4/IL‐36R, MyD88/NF‐κB, and p38 MAPK pathways in driving psoriasis‐induced skin inflammation and acute lung injury, respectively.^111^, ^112^ In rheumatoid arthritis (RA) patients, however, IgA autoantibody complexes can induce joint damage by activating FcαRI to induce the formation of NETs and increase the severity of the disease.^113^ In addition, studies have found that targeted blockade of NOX2 and p38 MAPK signaling can improve postoperative liver function and survival in patients with chronic liver disease caused by NETs.^114^ Inhibition of the activation of STAT1, HMGB1–TLR2/TLR4, PI3K‐γ, Raf1–MEK‐1–Erk can alleviate the clinical symptoms of various diseases caused by NETs such as SLE, RA), microscopic polyangiitis (MPA), and so on.^115^, ^116^, ^117^, ^118^ In summary, targeting and inhibiting the activation of signaling pathway proteins can treat NETs‐related diseases to a certain extent.

### 4.6 Autophagy is involved in the formation of NETs

Autophagy is highly conserved in neutrophils, not only involved in the differentiation of neutrophils in the bone marrow, but also plays an important role in neutrophil granule formation, degranulation, cytokine production, and NETs release. ANCA‐induced autophagy can promote the formation of NETs, which can be enhanced by the autophagy inducer rapamycin and attenuated by the autophagy inhibitor 3‐methyladenine (3‐MA).^119^ Ullah et al.^120^ found that pneumococcus induced neutrophil autophagy in a type III PI3K‐dependent manner. The apoptosis rate of human neutrophils stimulated by antilysosomal membrane protein‐2 antibody (anti‐LAMP‐2 antibody) was significantly reduced, and this effect was attenuated by autophagy inhibitors 3‐MA and LY294002. The apoptosis inhibitor ZVAD‐fmk and necrosis inhibitor NEC‐1 had no significant effect.^120^ Moreover, in neutrophils stimulated by anti‐LAMP‐2 antibodies, the ratio of LC3BI conversion to LC3BII significantly increases, accompanied by extensive vacuolization exhibiting typical autophagic features, demonstrating the involvement of autophagy in the formation of NETs in humans.^121^ Neutrophils and eosinophils from ATG5‐deficient mice fail to form NETs upon physiological activation or exposure to low concentrations of PMA.^122^ Studies have shown that NETs rely on autophagy and participate in the pathogenesis of pancreatic cancer,^123^ and the stress response protein REDD1 is involved in SLE end‐organ damage and fibrosis by regulating autophagy to promote NET formation.^124^ Therefore, inhibiting autophagy production may potentially play a role in the prevention and treatment of NETs‐related diseases.

### 4.7 Interaction of NETs with other programmed cell death

Apoptosis (type I cell death) is a tightly regulated form of programmed cell death (PCD) that triggers self‐destruction without external influence, characterized by distinct morphological changes and activation of specific caspases and mitochondrial pathways.^125^ Recent studies indicate that apoptosis and NETosis in neutrophils occur sequentially, with apoptosis preceding NETosis. When apoptotic neutrophils are not cleared in time, a secondary death process, NETosis, is initiated. In essence, neutrophil apoptosis leads to NETosis. Notably, research has shown that various cell death pathways (pyroptosis and necrosis) converge on NETosis in neutrophils.^126^

Pyroptosis, a novel form of PCD, relies on the Gasdermin family proteins forming membrane pores, causing cell swelling and membrane rupture, leading to the release of cellular contents and triggering an inflammatory response.^127^ Recent findings reveal that during sepsis, microvesicles (MVs) from pyroptotic macrophages accelerate NET formation through GSDMD‐N mitochondrial transfer. These MVs increase ROS production in neutrophils and induce DNA oxidation. Moreover, neutrophils undergoing NETosis engage in crosstalk within the microenvironment, leading to a cascade of responses.^128^

NETs contribute to the apoptosis, ferroptosis, and pyroptosis of target cells. For instance, NET‐derived MPO and H2O2 activate the NFκB signaling pathway through epithelial Toll‐like receptors (TLRs), promoting the inflammatory and angiogenic response of ECs by upregulating ICAM1, VCAM1, PECAM1, and increasing the secretion of IL6, IL8, and VEGFA. Additionally, NETs induce M1 macrophage polarization, influencing neutrophil phagocytosis and further NET formation.^129^ Neutrophil NETosis impacts ECs (increasing vascular permeability), macrophages (M1 polarization), and the inflammatory response of neutrophils themselves.^130^

Ferroptosis, a unique form of cell death driven by iron‐dependent lipid peroxidation, is regulated by various metabolic pathways, including redox balance, iron metabolism, mitochondrial activity, and amino acid, lipid, and glucose metabolism, along with disease‐associated signaling pathways.^131^ ROS is a shared critical factor in both ferroptosis and NET formation. Recent studies show that NETs promote intestinal microvascular endothelial ferroptosis by impairing mitophagy.^132^ Excessive NETs damage the endothelial glycocalyx, disrupting the SDC‐1/HS/HGF complex and hindering downstream cMET signaling, inducing endothelial ferroptosis and exacerbating tissue damage.^133^

**FIGURE 6:**
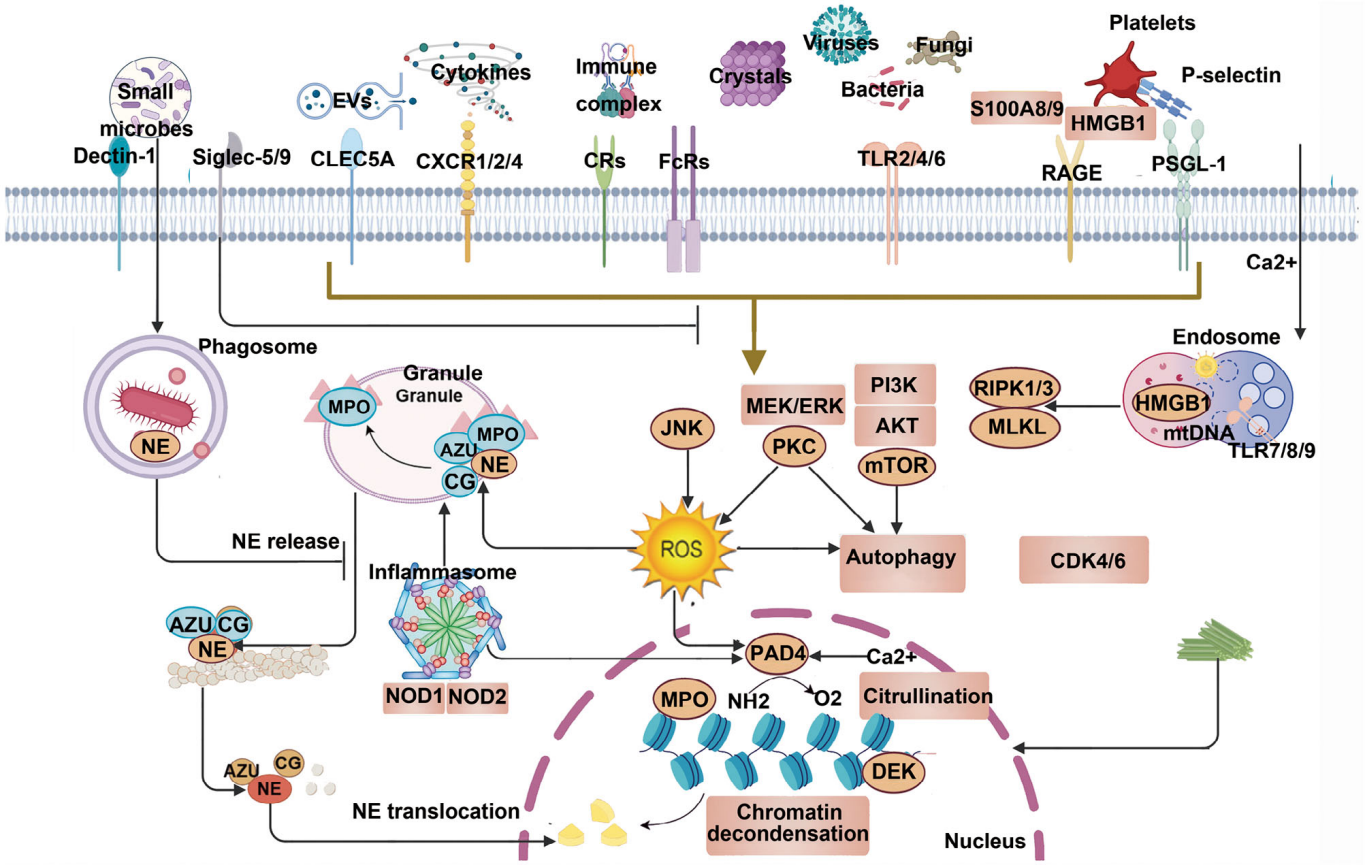
Interaction of NETs with other PCD. (Neutrophil signaling pathways are tightly regulated to ensure accurate chemotaxis. The PI3K pathway, activated by GPCRs, drives actin polymerization and cell polarization, with PTEN and SHIP balancing PI3K activity. The ERK pathway shapes cell polarity and cytoskeletal reorganization through Ras–Raf–MEK–ERK signaling. Crosstalk between PI3K and ERK fine‐tunes chemotaxis, while p38 MAPK, activated by terminal chemoattractants like fMLF, regulates actin dynamics. The JAK/STAT pathway mediates cytokine effects, with type I interferons enhancing antiviral and antitumor functions by modulating neutrophil lifespan and recruitment. Created by BioRender.)

Therefore, future drug development targeting various aspects of NETs may offer novel therapeutic approaches for NET‐related diseases, such as cardiovascular diseases, cancer, and autoimmune disorders. The mechanism of interaction of NETs with other PCD was summarized in Figure 6.

## 5 DETECTION TECHNIQUES OF NETs

Visualization methods for detecting NETs^135^, ^136^ mainly include electron microscopy (transmission electron microscopy and scanning electron microscopy), fluorescence microscopy, and time‐lapse video microscopy (live cell imaging). Among them, electron microscopy is a commonly used tool for characterizing the characteristics of NET formation. However, electron microscopy staining for NETs is not specific enough to differentiate NETs from host cell debris or other proteins present at inflammatory sites.^137^ Quantitative methods for detecting NETs mainly include flow cytometry, ELISA, and fluorescence microscopy.^138^ High‐speed multispectral imaging flow cytometry can assess the release of mitochondrial DNA to form NETs. ELISA can quantitatively detect the levels of citrullinated histone 3 (CitH3) in human plasma or the levels of NET‐specific MPO–DNA and NE–DNA complexes.^139^, ^140^ Quantification of NET formation can be achieved through fluorescence microscopy using different techniques, specific markers, and standardized assessment tools. This is also the most reliable method to differentiate NETosis from other cell death mechanisms such as apoptosis or necrosis. Lood et al.^141^ conducted a more in‐depth study on the origin of DNA in NETs while revealing the mechanism of mROS action. Through laser confocal microscopy and immunohistochemical techniques, it was confirmed that ICs containing ribonucleoprotein were prevalent in patients with SLE, and the NETs induced mainly consisted of mitochondrial DNA.^142^ Therefore, the detection and dynamic changes of NETs can further explore their molecular mechanisms, predict the progression of diseases, and provide personalized treatment references for patients with a high incidence of NETs. Based on previously reported NETs detection methods, they can generally be classified into qualitative and quantitative categories. Qualitative detection commonly employs electron microscopy techniques such as scanning electron microscopy, transmission electron microscopy, and delayed video microscopy to observe the formation of neutrophil NETs.^143^, ^144^ Morphological methods have subjectivity in judging the process, but they are convenient, quick, and can be used for simple qualitative assessments. From the perspective of network markers, electrophoresis techniques, fluorescent staining, confocal microscopy, flow cytometry, microplate staining, and fluorescent immunochromatography can be used for the detection of free DNA.^135^ Enzyme‐linked immunosorbent assays, Western blotting, flow cytometry, microplate staining, fluorescence immunochromatography, and other techniques can be used to measure protein components such as MPO and NE.^13^ However, each of these methods has its advantages and limitations, and a combination of multiple methods can be used for analysis. Quantitative detection methods are currently commonly used and can be divided into two types. One type is to evaluate the activity of NEs related to DNA by targeting citrullinated histones using an enzyme‐linked immunosorbent assay and estimating the coverage area of NETs through image analysis.^145^ The limitation of this strategy is the high false‐positive rate, as DNA release can also occur in processes such as necrosis. The other type is to evaluate the percentage of meshed nuclei in mixed cell populations based on distinguishable changes in nuclear shape (such as DNA adhered to fluorescently labeled neutrophils, flow cytometry, etc.).^146^ For the second strategy, researchers have used both adherent and nonadherent neutrophils for testing. Increasing evidence suggests that neutrophil adhesion is an important component of NETosis.^147^ Some inducers require neutrophils to adhere to the matrix before NETosis occurs. Therefore, a potential drawback of detection based on flow cytometry is that it is difficult to determine the number of NETosing cells. Elsherif et al.^148^ introduced machine learning algorithms into the classification and quantification of nuclear images of peripheral blood neutrophils, and evaluated them using convolutional neural networks (CNNs). CNNs achieved an accuracy of over 94% in distinguishing meshed nuclei from nonmeshed nuclei, significantly improving the analytical capabilities and screening levels of dose–response relationships in neutrophils for patients. Since NETosis and necrosis can be differentiated based on nuclear morphological features, as well as the distinct signal pathways of NETosis, CNNs may become a powerful tool for NETosis detection.

## 6 MECHANISMS OF NETS INVOLVED IN AUTOIMMUNE AND AUTOINFLAMMATORY DISEASES

In autoimmune and autoinflammatory diseases, NETs are considered potential disruptors of self‐tolerance, serving as reservoirs of autoantigens that promote autoantibody (AAb) production. NETs play a critical role in the progression of autoimmune diseases such as SLE, Behçet's disease, RA, ANCA‐associated vasculitis (AAV), and APS.^149^ SLE and monogenic systemic autoinflammatory diseases are often regarded as representative examples of autoimmune and autoinflammatory diseases, respectively.^150^

Autoimmune diseases are characterized by a loss of immune tolerance, recognition of self‐antigens, activation of T and B cells, and subsequent production of AAbs. This dysregulated adaptive immune response results in multiorgan damage. In contrast, autoinflammatory diseases are not antigen specific; they manifest as chronic systemic inflammation without compromising immune tolerance or generating specific AAbs. NET components further exacerbate the inflammatory milieu by promoting complement activation and activating immune cells such as B cells and antigen‐presenting cells, thereby sustaining autoimmune responses. Additionally, NETs act as mediators of platelet activation and coagulation, and their dysregulation or excessive formation contributes to pathological thrombosis in autoimmune diseases (e.g., SLE and APS) and autoinflammatory conditions (e.g., Behçet's disease).^151^ Moreover, NETs play a significant role in the complications of autoimmune diseases, including cardiovascular and reproductive immune disorders, further aggravating disease pathology.

### 6.1 Systemic lupus erythematosus

**FIGURE 7:**
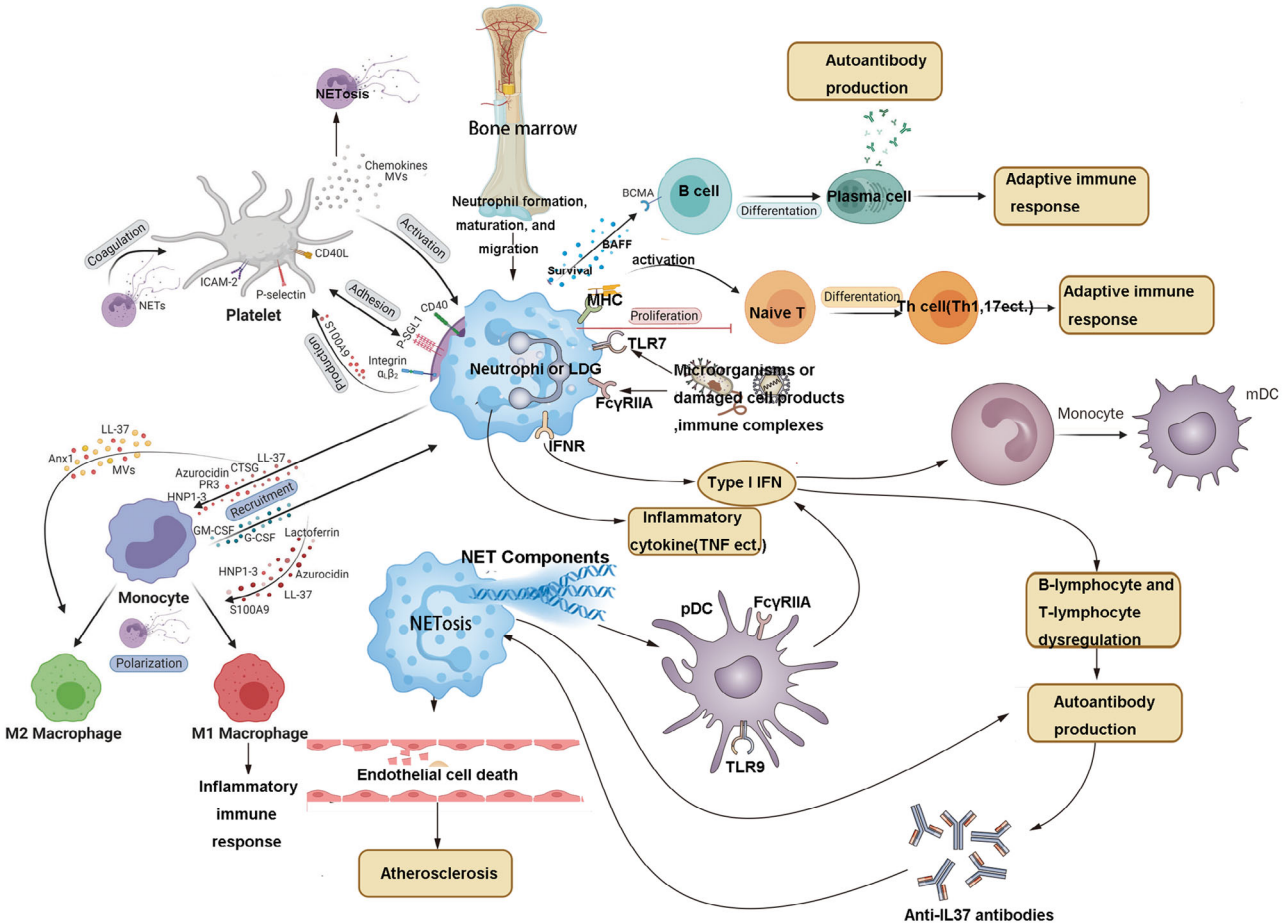
Mechanisms of neutrophil extracellular traps involved in SLE. (Neutrophils in SLE patients exhibit reduced phagocytic capacity, impaired ROS production, and excessive NET formation. NETs, composed of dsDNA, histones, and proteins, are key dsDNA antigen sources and deposit in skin and kidney tissues, inducing pDC‐driven type I IFN production. Low‐density neutrophils, prone to spontaneous NET formation, contribute to endothelial damage and vascular lesions, though their therapeutic targeting remains unclear. Created by BioRender.)

SLE is a typical autoimmune inflammatory connective tissue disease, which can present as multisystem and multiorgan involvement.^152^ The etiology and pathogenesis of SLE are not yet fully understood, and the development of the disease is influenced by genetic factors (possibility of one or multiple disease‐susceptibility genes), immune, neuroendocrine, and environmental factors (such as ultraviolet radiation, drugs, viral infections).^153^ Among the immunological factors, self‐antibodies generated against double‐stranded DNA (dsDNA) and other nuclear components play a crucial role.^154^ The production of such antigens in the body is usually associated with impaired apoptosis cell clearance, leading to apoptotic cell extravasation and the release of self‐antigens. These self‐antigens are presented by follicular dendritic cells in secondary lymphoid organs to activate autoreactive B lymphocytes and prolong their lifespan, obstructing negative selection and leading to abnormal immune tolerance in peripheral circulation.^155^ The activation of autoreactive B lymphocytes and the production of AAbs constitute the first stage of the pathogenesis of SLE, and the second stage is the in situ and/or ectopic deposition of ICs formed by self‐antibodies and self‐antigens, leading to tissue damage, mediated by phagocytes that engulf ICs and release proinflammatory factors^156^ (Figure 7).

#### 6.1.1 Mechanisms of neutrophil involvement in SLE

In patients with SLE, there is a large number of AAbs in the body. The ICs inside the tissues require the recruitment of neutrophils from the peripheral circulation.^157^ Neutrophils in SLE exhibit a heightened propensity to form NETs, partly due to their strong response to elevated levels of type I interferons (IFN‐I). When activated by various stimuli, including RNA ICs, neutrophils—particularly low‐density granulocytes (LDGs)—can secrete cytokines and generate NETs. The impaired clearance of NETs by DNase leads to an accumulation of substrates for IC formation, further amplifying inflammation. Neutrophils and NETs play a pivotal role in vascular damage mediated by the innate immune system in SLE, contributing significantly to disease pathology.^158^, ^159^ The phenotype and function of neutrophils in individuals with lupus are also unique. LDGs, which have been isolated from lupus patients, are more prone to activation and exhibit enhanced proinflammatory functions.^160^ Therefore, the response of neutrophils to FcγR stimulation with active disease or the recruitment of neutrophils to tissues is a matter of interest for investigation.

Previous studies have reported abnormalities in neutrophil apoptosis and the clearance of apoptotic bodies in SLE patients, which are correlated with disease activity.^161^ Furthermore, SLE patients exhibit an increased proportion of neutrophils undergoing secondary necrosis.^161^ As the loss of these cells increases, neutropenia, which is characterized by a decrease in circulating neutrophil numbers, becomes a common symptom among SLE patients.^162^ The large number of neutrophils and their shorter lifespan compared with other blood cell types account for these observations, suggesting that the increased neutrophil apoptosis may be associated with the pathological mechanism underlying SLE onset. Therefore, the abundant apoptotic neutrophils may serve as a source of antigenic material, leading to the overproduction of AAbs in SLE patients, including antibodies against dsDNA and nuclear histones.^163^ The mechanisms driving neutropenia in SLE patients include the removal of neutrophils driven by AAbs, neutralization of growth factors acting on neutrophils (such as G‐CSF), bone marrow suppression, increased neutrophil pyroptosis and secondary necrosis, and death caused by NETosis.^164^

Moreover, the enhanced apoptosis and abnormal clearance of neutrophils after apoptosis contribute to neutropenia through different mechanisms, including reduced expression of CD44 and increased expression of FAS, as well as the production of AAbs against dsDNA.^165^ ANCAs have also been found in SLE patients.^164^ The detection of ANCA in the serum of pediatric patients with SLE revealed a series of antibodies against intracellular proteins of neutrophils, including MPO, lactoferrin, human NE, and neutrophilic extracellular traps.^163^ However, these specific antigens were not found to be associated with disease activity or target organ involvement. The cross‐reactivity of AAbs targeting SSB/La nuclear proteins can bind to specific sites on neutrophil membrane proteins.^166^

Additionally, neutrophils derived from SLE patients often display an increased amount of membrane‐bound immunoglobulins, leading to binding with antineutrophil antibodies and IC deposition. These findings suggest that AAbs contribute to the symptoms of neutropenia in SLE patients and are consistent with clues from antibody‐mediated autoimmune neutropenia in other autoimmune diseases. AAbs specific to neutrophil precursors have been identified in patients with autoimmune neutropenia, and this observation may be a contributing factor to the development of SLE, as measured by colony‐forming assays detecting AAbs in vitro from patients with the disease.^167^ The observation that a subset of neutrophils infiltrating organs may be actively undergoing NETosis or apoptosis suggests a possible explanation for the occurrence of neutropenia in patients, which may also lead to organ damage and immune dysregulation.^168^ However, this theory requires further investigation and more evidence to support it. In summary, neutrophils play a key role in the pathogenesis of SLE induced by ICs. Circulating neutrophils can function as effector cells in the presence of circulating ICs or as secondary effector cells after recruitment to tissues. Neutrophils can directly react to deposited ICs or indirectly respond to the activation of resident cells by ICs. The activation of neutrophils in tissues or circulation can lead to different biological responses. Most neutrophils can directly cause tissue damage and can induce injury when injected into animals. SLE patients also exhibit notable abnormalities in the phenotype and function of neutrophils, with enhanced death by apoptosis and NETosis, which may also serve as a source of autoantigens. The abnormal neutrophils with enhanced proinflammatory function in the presence of large amounts of AAbs in the peripheral circulation and tissues of SLE patients indicate the significance of further research on neutrophils for the progression of SLE.

#### 6.1.2 Mechanisms of NETs in SLE

Studies have shown that SLE patients can produce DNAse I inhibitors or antibodies specifically targeting NETs, resulting in a decreased ability to degrade NETs and further exacerbating the progression of SLE.^169^ In addition, complement activation in SLE patients can bind to incompletely degraded NETs, leading to a reduction in degradation capacity, which is one of the reasons for reduced degradation ability.^170^ It has been confirmed that NETs are deposited and activate complement in SLE tissues, further amplifying the inflammatory response.^170^ Courtney et al.^171^ showed that SLE patients have an increased number of neutrophils prone to spontaneous cell death in the peripheral blood, along with an increase in levels of dsDNA antibodies, which are positively correlated with the severity of SLE development. Lande et al.^172^ found that ICs formed by neutrophil granule peptide LL37 and dsDNA in the serum of SLE patients can activate plasmacytoid dendritic cells (pDCs) through TLR9 receptors and act as autoantigens to activate B cells, indicating an important link between the activation of NETs, pDCs, and autoimmunity. Jiang et al. collected fasting peripheral venous blood from 44 female SLE patients and used fluorescence brightness assay to quantitatively measure the formation rate of NETs. The results showed that the NETs level in female SLE patients was significantly higher than that in the healthy control group, indicating an enhanced formation of NETs in SLE patients. Moreover, the level of NETs in SLE patients was positively correlated with the SLE disease activity score, suggesting that NETs may serve as an evaluation marker for the severity of SLE.^173^ In conclusion, a large body of evidence has demonstrated the important role of NETs in the occurrence and development of SLE.

### 6.2 Lupus nephritis

The pathogenesis of lupus nephritis (LN) is mainly associated with the deposition of ICs formed by self‐antigen‐antibody complexes in the glomerulus, renal tubules‐interstitium, and blood vessels, which belong to IC glomerulonephritis. Among them, nearly half of SLE patients develop LN in the early stage of the disease, and nearly one‐third progress to end‐stage renal disease.^174^, ^175^ Wang et al.^176^ for the first time captured the dynamic process of neutrophils releasing DNA in the glomerular capillaries of live lupus mice using two‐photon intravital imaging, which provides a theoretical basis for the clinical development of GSDMD inhibitors targeting DNA release in the treatment of SLE. Han et al. found that the level of NETs is associated with the severity of LN renal injury and the occurrence of renal endpoints, thus serving as an indicator for the prognosis of LN.^177^ In addition, Hakkim et al. found a correlation between impaired NETs clearance and the occurrence of SLE kidney disease.^178^, ^179^ In terms of mechanisms, Skopelja‐Gardner et al.^180^ found that the migration of neutrophils expressing CXCR4 and intercellular adhesion molecule 1 (ICAM1) is attracted to the local kidney by the chemoattractant factor CXCL12 produced by podocytes and renal tubular epithelial cells. Although other inflammatory cells may also participate in the process of inflammation expansion from the skin to the kidneys through interstitial migration, they do not migrate as rapidly as neutrophils. However, it is still unclear why the expression of CXCL12 increases locally in the kidney and why neutrophils do not migrate to other organs such as the lungs. It is worth noting that the migration of neutrophils from the skin to the kidneys also depends on granulocyte colony‐stimulating factor G‐CSF.^180^ Blocking G‐CSF is accompanied by a decrease in the expression of adhesion molecules (VCAM1 and E‐selectin), proinflammatory cytokines (IL‐1β), and tissue damage‐related molecules (NGAL and HAVcr‐1) in the kidney. However, there are also studies reporting that G‐CSF can amplify the regulatory T cells and alleviate LN, so there is controversy over the role of G‐CSF in lupus, and further research is needed to determine the therapeutic value of blocking this cytokine in SLE. SLE patients also have an increased number of LDG clusters in the peripheral blood.^181^ A study confirmed that similar phenotypic neutrophils (i.e. CXCR4hiICAM1hiCR1lo) are also present in the peripheral blood of lupus mice exposed to ultraviolet radiation. Therefore, it is still unclear whether continuous exposure to ultraviolet radiation causes an increase in LDG in the peripheral blood of SLE patients, but this is worth further investigation. This study for the first time revealed cell exchange between two independent organs, which is of great significance for elucidating the multiple organ damage in SLE.^182^ In our previous study, we found that neutrophils from active SLE patients exhibit spontaneous ferroptosis, which can be restored to normal levels after effective treatment. Autoimmune reactive IgG and IFN‐I can induce ferroptosis in neutrophils. Ferroptosis inhibitors can significantly alleviate the progression of lupus in mice. The deletion of the ferroptosis negative‐regulating protein GPX4 specifically in the myeloid lineage in mice can induce a typical lupus‐like phenotype. Autoimmune reactive IgG and IFN‐I inhibit the expression of GPX4 in SLE patients’ neutrophils through the CaMKIV/CREM pathway, leading to the accumulation of intracellular lipid ROS and neutrophil ferroptosis.^183^

### 6.3 Autoimmune vascular inflammation

#### 6.3.1 Mechanisms of NETs in endothelial dysfunction in Behcet's disease

Behcet's disease (BD) is a chronic systemic vasculitis characterized by recurrent lesions in the skin, mucous membranes, eyes, gastrointestinal tract, and brain. This immune‐inflammatory disease involves various types of blood vessels and different sizes of the vascular tree and is often complicated by recurrent thrombosis, especially in the venous sinuses.^184^, ^185^ The presence of chronic inflammation in BD suggests increased oxidative stress, inducing the release of proinflammatory cytokines and chemokines that activate platelets, leukocytes, and ECs. Overactivated neutrophils in BD exhibit increased phagocytic activity and superoxide generation,^186^ which may contribute to the formation of fibrinogen oxidation clots.^187^ Hyperactivation of neutrophils is considered the basis of BD.^188^, ^189^ Flow cytometry analysis has revealed the activation of neutrophils in active BD cases.^190^ Similarly, histopathological examination of skin, synovium, intestinal, and central nervous system (CNS) biopsy tissues confirms the infiltration of neutrophils.^191^, ^192^ A recent study integrating multiomics data on BD revealed that neutrophil metabolic abnormalities may contribute to disease pathogenesis. Through intervention experiments, the study identified a key metabolite, farnesyl pyrophosphate, as a critical factor. Further analysis demonstrated that TNF‐α induces the expression of TRPM2 channels in neutrophils, a process that can be effectively blocked by classical TNF inhibitors. This intervention mitigates excessive neutrophil activation and vascular inflammation in BD, highlighting a potential therapeutic strategy for managing the disease.^187^ All these data support the crucial role of neutrophils in BD pathophysiology. In a recent study, NETs from BD patients have been shown to induce endothelial dysfunction by reducing cell proliferation and increasing cell apoptosis, suggesting a role of NETs beyond thrombosis.^193^ Interestingly, recent findings indicate that treatment methods widely used in BD, such as colchicine, anti‐TNFα, or anti‐IL‐6, can inhibit the release of NETs in vitro and further alleviate endothelial dysfunction and immune cell activation, thereby affecting the overall activity of the vascular system. Among them, neutrophil‐mediated mechanisms may directly contribute to the pathogenesis of Behcet's syndrome, and NETs may be more involved in the mechanisms underlying mucosal, joint, and intestinal manifestations than ROS. Colchicine may effectively counteract neutrophil‐mediated damage in BD, but further research is needed.^194^ In the future, potential therapeutic targets to reduce circulating NETs in BD may include ROS scavengers like N‐acetylcysteine, PAD inhibitors like Cl‐amidine and DNase used in SLE^195^, ^196^ and RA, which have been shown to be effective.^197^, ^198^

Our preliminary research showed that NETs production in neutrophils from BD patients was higher than in neutrophils from healthy controls.^199^ Both serum NETs from BD patients and NETs released by BD neutrophils had higher levels of oxidized DNA than those from healthy controls.^199^ Recent findings by Mariam et al. suggested that the neutrophil population in BD is heterogeneous, and the increased number of LDNs may contribute to inflammation and pathogenesis.^200^, ^201^, ^202^ Alexandre et al. found that levels of NETs and NETs markers were elevated in BD patients and contributed to a procoagulant state.^203^ Targeting NETs may represent a potential therapeutic target for reducing or preventing the risk of thrombosis associated with BD.

#### 6.3.2 NETs involved in vasculitis and thrombosis in BD

Safi et al.^204^ found that neutrophils contribute to vasculitis in Behcet's disease by increasing the release of NETs. Additionally, ECs cultured in the presence of NETs from BD patients exhibited reduced proliferation and increased apoptosis and cell death, indicating endothelial dysfunction.^204^ Kawakami et al.^205^ investigated the association between NETs and isolated superficial venous thrombophlebitis in three BD patients by immunostaining for MPO and histone H3 citrullination (His‐3‐Cit) protein in skin biopsy tissues, and found the presence of NETs in neutrophilic infiltrates and lipogranulomatous inflammation surrounding the MSU crystals, as well as in superficial venous thrombophlebitis. The role of NETs in thrombosis has been established in animal models of venous thrombosis^206^ and human studies,^207^ revealing the specific role of NETs in the pathogenesis of thrombosis. Thus, the pathogenesis of BD‐associated thrombosis may be related to neutrophil activation, and the release of NETs contributes to the development of lipogranulomatous inflammation in affected skin lesions and nodular lesions.

### 6.4 Gout/gouty arthritis immune inflammation

#### 6.4.1 NETs promote inflammatory reactions in GA

Gout/gouty arthritis (GA) is an inflammatory disease caused by the deposition of monosodium urate (MSU) crystals.^208^ When the uric acid concentration in the body exceeds its solubility and reaches a supersaturated state, MSU crystals deposit in cartilage, synovium, and surrounding tissues, stimulating the synovium and triggering a series of pathological reactions, leading to acute inflammation in the joints.^209^ Activation of neutrophils by MSU crystals can induce the production of NETs, which in turn capture and phagocytose the MSU crystals and exert their antimicrobial actions with embedded enzymes such as elastase, MPO, and tissue protease G.^210^, ^211^

In GA, a large amount of IL‐1β is produced and released when the MSU crystals activate the NLRP3 inflammasome and bind to receptors on neutrophils and macrophages, leading to the activation of the adaptor protein MyD88.^212^ The emerging role of NETs in autoimmune and autoinflammatory diseases highlights a complex inflammatory response, where NETs play a critical part in disease progression. In the context of acute GA (AGA), stimulated by MSU crystals, NLRP3 inflammasomes are formed, leading to the production and release of proinflammatory cytokines, such as IL‐1β, which amplifies inflammation and recruits neutrophils to the site of injury, intensifying joint pain.^208^ Neutrophils, upon engulfing MSU crystals, are induced to form NETs, which contribute to the host's defense.^209^ As NETs are generated, neutrophil DNA, histones, and inflammatory mediators spill into the extracellular space, triggering immune system activation. During NETosis, ATP released from neutrophils enters the synovial cavity, acting as a stimulus for P2×7 receptors. This receptor mediates potassium efflux, activating IL‐1β‐converting enzyme, which promotes IL‐1β maturation. Concurrently, it triggers NLRP3 inflammasome activation, where caspase‐1 cleaves pro‐IL‐1β into its active form, releasing mature IL‐1β,^213^, ^214^ further promoting oxidative bursts in neutrophils and NET formation. As cell damage occurs, uric acid levels in the joint cavity rise, exacerbating inflammation, which in turn causes more crystal precipitation and amplifies the inflammatory response. The role of the ROS–NLRP3 inflammasome in MSU crystal‐induced inflammation is still not fully understood. However, it has been demonstrated that the activation of NLRP3 inflammasomes requires thioredoxin‐interacting protein, which regulates the redox state of the cell and mediates the nuclear factor‐kappa B signaling pathway.^215^ Another study indicates that the NLRP3‐dependent release of IL‐1β is modulated by caspase‐1 activation through PI3Kγ. In wild‐type mice injected with MSU crystals into the knee joint, the absence or inhibition of PI3Kγ reduced IL‐1β levels, neutrophil recruitment, and joint pain.^216^ Caution et al.^217^ first proposed that caspase‐11 mediates neutrophil migration and NET formation during AGA by altering the phosphorylation of cofilin, an actin‐regulating protein. This process is closely linked to caspase‐1 activation and the subsequent release of IL‐1β. This study sheds light on the intricate interactions between NETs, NLRP3 inflammasomes, and other inflammatory pathways in disease mechanisms, particularly within autoimmune and autoinflammatory diseases.^217^ These findings offer potential targets for therapeutic interventions aimed at mitigating the harmful effects of excessive inflammation.^218^, ^219^

The formation of NETs by neutrophils in response to MSU crystals can further capture and eliminate invading pathogens, enhancing the antimicrobial activity. The adhesion of crystals to NETs is similar to the capture of bacteria. Aggregated NETs induced by the aggregation of MSU crystals, called aggNETs, can reverse‐capture inflammatory factors and MSU crystals, degrading the trapped inflammatory factors and chemokines through the serine protease enzymes embedded in the NETs, breaking the vicious cycle of inflammation.^220^ This suggests that NETs also play an important role in the spontaneous resolution of GA.^220^ Neutrophils recruited to the site of inflammation can also promote the production of transforming growth factor‐β1 through NET formation, which inhibits neutrophil oxidative bursts and acts as a nonessential regulator of IL‐1β maturation, reducing NET generation.^221^ It is considered an important anti‐inflammatory factor in the spontaneous resolution of gout and works together with resolving and protecting factors to promote the self‐resolution of inflammation.^222^

#### 6.4.2 Characteristic structure of NETs in gouty tophi formation

Recent studies have shown that the embedding of MSU crystals within aggNETs may be the basis for tophi formation and may influence the progression of the disease.^223^ aggNETs and MSU crystals interact with each other for a long time, forming a complex multilayer structure that gradually develops into visible tophi.^224^ Chatfield et al.^225^ demonstrated that NETs induced by MSU crystals and other physiologically relevant crystals have a different molecular pathway than those induced by PMA and Candida, making them resistant to degradation by DNase I. This implies that MSU crystals enveloped by aggNETs are more difficult to dissolve and clear, leading to the formation of chronic tophaceous gout. Furthermore, several studies have reported that certain natural compounds and herbal formulations may alleviate gout inflammation by reducing NETs, such as quercetin and Si‐Miao‐Tang.^226^, ^227^, ^228^, ^229^, ^230^ In the future, a deeper understanding of NETs can accelerate the research on targeted drugs related to NETosis and lay the foundation for the intervention of gout.

### 6.5 Rheumatoid arthritis

RA is a common chronic autoimmune disease, with an average global prevalence of 1% and a prevalence of 0.3–0.4% in China.^231^ RA is characterized by synovial inflammation, synovial hyperplasia, and angiogenesis, leading to bone and cartilage destruction, resulting in joint pain, swelling, stiffness, and functional impairment.^232^ A significant number of RA patients develop RA‐related AAbs, including rheumatoid factor (RF) and AAbs to citrullinated protein antigens (ACPAs).^233^ The formation of ACPAs is considered a key pathogenic event, as ACPAs can be detected in the serum of RA patients 3–5 years before the onset of clinical symptoms.^234^ In recent years, research has found a close relationship between NETs and ACPAs, playing a key role in various autoimmune diseases, including RA. It has been discovered that NETs are an important source of citrullinated proteins and cytokines in RA, and they are also a major source of ACPAs.^157^

In addition to the activated phenotype in peripheral blood, a large number of activated neutrophils have been found in the synovial joints and tissues of RA patients.^235^, ^236^, ^237^, ^238^, ^239^, ^240^ A recent study published in Nature revealed that the absence of the myeloid inhibitory C‐type lectin‐like receptor (MICL) accelerates the progression of RA by promoting NET formation.^241^ The presence of activated neutrophils in the joints of RA patients is accompanied by high levels of neutrophil granule proteins in the synovial fluid (SF).^242^, ^243^, ^244^, ^245^, ^246^ These granule proteins promote the pathogenesis of RA by protein hydrolysis, activation of signaling proteins (including cytokines and chemokines), cleavage of soluble receptors (such as IL‐6 receptor), and degradation of cartilage (such as collagen degradation).^247^, ^248^, ^249^, ^250^ Neutrophils in the SF exhibit a higher level of superoxide production and contain phosphorylated p47phox, indicating the assembly and activation of NOX.^251^ They also express high‐affinity FcγRI receptors (CD64) and major histocompatibility complex II (MHC II) proteins.^252^, ^253^, ^254^, ^255^ The NE levels in the SF are lower, indicating that they have undergone degranulation. Early animal studies and human case studies have shown that neutrophils play an important role in the initiation of synovial inflammation in RA^241^, ^256^ and may promote synovial fibroblast adhesion and proliferation by releasing granule enzymes and vascular endothelial growth factor (VEGF).^257^, ^258^ It has been proposed that citrullinated antigens play a key role in NETs formation, initiation of autoimmunity, and the development of ACPAs in RA.^259^ NET products have been detected in serum and SF of RA patients,^260^ and NETs have been observed in RA synovial tissue by immunohistochemical staining for CD15, elastase, MPO, and CitH3.^259^, ^261^ Recent studies have shown that up to 70% of newly diagnosed RA patients have serum AAbs that recognize NET components (ANETAs).^262^ Helen et al. found that neutrophils in RA SF drive inflammation through the production of chemokines, ROS, and NETs. In addition, they found that RA SF neutrophils lose their migratory properties, stay in the joints, and signal to promote joint damage by recruiting and activating innate and adaptive immune cells.

#### 6.5.1 NETs as a source of citrullinated proteins and cytokines in RA, stimulating the production of ACPAs

RA patients have a significant number of neutrophils in the SF. When neutrophils are exposed to various stimuli (such as cytokines, TLR ligands, etc.), they are activated, leading to extensive protein citrullination.^264^, ^265^, ^266^ Neutrophils in the SF of RA patients have been found to have an abnormal phenotype, with increased production of ROS and higher phosphorylation of p47(phox), indicating the assembly and activation of NOX2.^251^ They also exhibit high expression of the FcγR1 receptor and MHC II molecules.^252^, ^253^, ^254^, ^255^ The SF neutrophil lysates have lower levels of granule proteins (such as MPO), suggesting that they have undergone degranulation in the synovial joints.^242^ Animal studies and human case studies of early‐stage RA have shown that neutrophils play an important role in the initiation of synovial inflammation^241^, ^256^ and may contribute to synovial fibroblast adhesion and growth through the release of granule enzymes and VEGF, promoting joint inflammation.^257^, ^258^ Neutrophils in the SF have altered phenotypes compared with paired blood neutrophils. They produce higher levels of superoxide and contain phosphorylated p47(phox), indicating the assembly and activation of NOX.^251^ They also express high‐affinity FcγR1 receptors and MHC II molecules.^252^, ^253^, ^254^, ^255^ The SF neutrophil lysates show lower levels of granule proteins (such as MPO), suggesting that they have undergone degranulation.^242^ Early studies in animals and humans have shown that neutrophils play a significant role in the initiation of synovial inflammation in RA.^241^, ^256^ They may promote synovial inflammation by releasing granule enzymes, such as MPO, and growth factors, such as VEGF. Neutrophils in the SF are found in large numbers and are accompanied by high levels of neutrophil granule proteins, including MPO, elastase, proteinase 3 (PR3), elastase, and lactoferrin.^242^, ^243^, ^244^, ^245^, ^246^ These granule proteins play a role in RA pathogenesis by protein hydrolysis, activation of signaling proteins (including cytokines and chemokines), degradation of soluble receptors (such as IL‐6 receptor), and cartilage degradation (such as collagen degradation).^247^, ^248^, ^249^, ^250^ In comparison with neutrophils in paired blood samples, neutrophils in SF exhibit altered phenotypes. They produce higher levels of superoxide and contain phosphorylated p47phox, indicating the assembly and activation of NOX2 in vivo.^251^ They also express high‐affinity Fcγ receptor 1 and MHC class II proteins.^252^, ^253^, ^254^, ^255^ Neutrophil‐derived products in SF have lower levels of granule proteins (such as MPO), indicating that they have undergone degranulation within the synovial joints.^242^ Animal studies of early RA and human case studies have shown that neutrophils play a crucial role in the initial synovial inflammation of RA.^241^, ^256^ This may occur via the release of granule enzymes and production of VEGF, both of which promote the adhesion and growth of fibroblast‐like synoviocytes (FLSs) in the inflamed synovial vessels.^257^, ^258^ The key role of citrullinated antigens in NETs has been proposed in the initiation of autoimmune responses and the development of ACPAs in RA.^259^ NET products have been detected in the serum and SF of RA patients.^260^, ^261^ Recent studies have also shown that up to 70% of newly diagnosed RA patients have serum AAbs recognizing NET components (ANETA). Wright et al.^262^ found that neutrophils in the SF of RA drive inflammation through the production of chemokines, ROS, and NETs. They also discovered that RA SF neutrophils lose their migratory properties and reside in the joints, producing signals that promote joint damage by recruiting and activating innate and adaptive immune cells.^262^

#### 6.5.2 NETs are a source of citrullinated proteins and cytokines that stimulate the production of ACPAs

SF in RA patients contains a large number of neutrophils, and when exposed to various stimuli (such as cytokines and TLR ligands), neutrophils are activated and undergo extensive protein citrullination.^264^ During infections or inflammatory stimuli, neutrophils release NETs to trap and kill pathogens, and protein citrullination is a key step in the formation of NETs. Approximately 70% of the proteins in NETs are histones, which are primarily derived from PADs in the mononuclear phagocyte system, catalyzing the conversion of arginine residues to citrullines in proteins.^265^ Additionally, the formation of pores in neutrophil membranes by perforin and complement membrane attack complex increases intracellular calcium levels and activates PADs.^266^ Therefore, the formation of citrullinated proteins in SF neutrophils can be induced by the formation of pore complexes, complement activation, and membrane attack complex, leading to extensive protein citrullination.

NET formation is enhanced in peripheral blood and SF of RA patients and is correlated with ACPA levels and joint inflammation.^264^ Neutrophils in RA SF, in the absence of microbial stimulation, are activated by RA inflammatory mediators ^266^ and can react with citrullinated proteins derived from NETs in RA serum,^264^ thus promoting the formation of NETs. Corsiero et al.^267^ further elucidated that the release of citrullinated proteins locally contributes to the expansion of high‐mutated B lymphocytes in ectopic lymphoid tissue in the synovium, leading to the production of large amounts of high‐affinity ACPAs, which exert an anti‐NET effect. Therefore, NETs may be a source of citrullinated antigens that exacerbate the autoimmunity in the synovium of RA. In recent years, some experimental data have proposed the opposite view, suggesting that there is no excessive citrullination in NETs.^266^, ^268^, ^269^ It is believed that the misclassification of citrullinated antigens has occurred in these cases, as they may originate from increased citrullination of damaged leukocytes due to mitochondrial autophagy defects. Although these NETs share morphological similarities (such as the release of cfDNA) with genuine NETs, they differ in their physiological functions. These processes can be induced by different stimuli that activate distinct biochemical pathways, thereby differentiating the occurrence of citrullination and antimicrobial action. The citrullinated autoantigens of RA may result from the exacerbation of leukocyte damage‐induced citrullination rather than NETs. Based on these studies, the role of NETs in citrullinated antigen generation should be reevaluated. This reevaluation should consider the stimuli used, as the investigation of the pathogenesis of RA often uses calcium ionophores to induce citrullination in neutrophils, which mimics the enhanced citrullination induced by leukocyte damage, rather than NETs. Meanwhile, gaining a deeper understanding of NETs can accelerate the research progress on targeted drugs related to NETosis, opening up new avenues for intervention in gouty disease.^269^, ^270^

#### 6.5.3 RA antibodies and inflammatory cytokines and SAA induce NET formation and create a vicious cycle

AAbs can be detected in the serum of patients with autoimmune diseases, including RA, including ANCA and antinuclear ribonucleoprotein antibodies. When neutrophils are activated by inflammatory cytokines, they create an inflammatory environment that is conducive to the induction of NET formation in the absence of microbial stimuli.^271^ Khandpur et al.^265^ also demonstrated that ACPAs, RF, and inflammatory cytokines such as TNF‐α and IL‐17 can enhance NET formation in RA. Conversely, the released citrullinated self‐antigens and various immune stimuli from NETs can trigger an autoimmune response and lead to the exacerbation of inflammation, forming a vicious cycle. Recent research^272^ has shown that serum amyloid A (SAA) is involved in the inflammatory mechanisms of RA through the induction of NET formation by neutrophils. Meng et al. found that serum amyloid P induces the formation of NETs in RA SF neutrophils via TLR4 signaling.^273^ Another study found that SAA induces NET formation through the SAA–TLR2–PI3K/Akt signaling pathway and directly contributes to the pathogenesis of RA arthritis.^274^ Darrah et al.^275^ found that 74% of the identified proteins in NETs are targets of autoimmune antibodies in autoimmune diseases, further indicating the important role of aberrant NET formation in RA.

#### 6.5.4 Activation of FLSs by NETs in RA contributes to synovial tissue hyperplasia

RA‐FLSs are the main effector cells in synovial hyperplasia in RA patients and play a significant role in attracting inflammatory cells to the synovium, promoting the transition from acute to chronic inflammation.^276^ IL‐17 has been found to promote TNF‐α‐induced IL‐1, IL‐6, and IL‐8 synthesis in synovial fibroblasts.^277^ Based on these studies, Khandpur et al.^265^ exposed synovial fibroblasts to NETs and found that the mRNA and protein levels of IL‐6 and IL‐18 were significantly upregulated, and further promoted NET formation. Treatment with deoxyribonuclease I (DNase I) significantly reduced this upregulation.^265^ Another study demonstrated that fibrinogen, an autoantigen identified in NETs, induces the release of inflammatory cytokines from RA synovial fibroblasts and enhances the expression of PAD4 and receptor activator of NF‐kB ligand in RA synovial fibroblasts, suggesting that autoantigens in NETs, such as fibrinogen, may play a critical role in activating and amplifying the inflammatory response in RA.^278^ These results suggest that NETs can promote the expression of proinflammatory genes in synovial fibroblasts and may amplify harmful inflammatory responses in the synovium of RA. However, the specific mechanisms involved require further investigation. Meng et al. found that there are more NETs present in RA synovial tissue and that low concentrations of NETs can promote angiogenesis.^263^ Carmona‐Rivera et al.^279^ found that NE, which is present in NETs, can directly degrade cartilage components in the synovium, and also amplify the proinflammatory pathways in synovial fibroblasts and macrophages, thereby exacerbating joint damage and creating a vicious cycle. Zhang et al. found that NETs promote the proliferation of RA‐FLSs.^280^ Additionally, NETs stimulated the downregulation of MST1 kinase expression in RA‐FLSs, and MST1 kinase can regulate immune responses, suggesting that NETs may be involved in the pathogenesis of RA through MST1‐mediated mechanisms.^281^ Recent studies have found that NETs are internalized by RA‐FLSs via a glycation end product receptor TLR9 pathway, leading to the production of proinflammatory cytokines by FLSs.^282^ MST1/2 kinases play important roles in the innate immune defense systems of mice and fruit flies and regulate the expression of inflammatory cytokines induced by TLRs.^283^

#### 6.5.5 PADs enzyme catalyzes protein citrullination in NETs

The PADs enzyme family has five subtypes, and neutrophils widely express PAD2 and PAD4.^284^ PADs enzymes catalyze the conversion of proteins into citrullinated antigens, a key step in the autoimmune response of patients positive for ACPAs in RA.^285^ Spengler et al.^286^ found that extracellular DNA in the SF of RA patients is closely associated with neutrophil concentration and PADs enzyme activity. Neutrophils release PADs enzyme activity in the supernatant after NET formation. During the progression of RA, neutrophils overexpress PADs, which leads to the accumulation of citrullinated proteins in the SF, serving as a source of extracellular self‐antigens in RA patients.^287^

#### 6.5.6 Abnormal clearance of NETs in RA

Immunofluorescence staining experiments of synovial tissue have confirmed that there are significantly more NETs present in the synovial tissue of RA patients compared with osteoarthritis (OA) patients. The clearance of NETs mainly relies on the degradation by DNase I and phagocytosis by macrophages. Meng et al.^287^ found that the inhibitory effect of DNase I on NET formation becomes stronger with increasing concentrations of DNase I. Xu et al. also found that the activity of DNase I is decreased in the serum and SF of RA patients, with even lower activity in the SF. The activity of DNase I in the SF of RA patients is negatively correlated with the number of neutrophils.^288^ In addition, the level of cfDNA in the SF is significantly higher than that in healthy controls, and there is a positive correlation between cfDNA in the SF and the number of neutrophils in RA patients.^19^, ^286^ The above studies suggest that NETs composed mainly of cfDNA may not be effectively cleared due to a decrease in DNase I activity. Certain pathogens can produce DNase I, which may reduce NETs’ ability to bind and kill self‐antigens by degrading the NETs.^289^ In RA, NET formation depends on ROS production in neutrophils, and DNase I may inhibit NET formation by inhibiting ROS generation and promoting the degradation of previously formed NETs. In addition to enzymatic degradation, macrophages can phagocytose NETs, leading to their clearance.^290^ Consol et al.^24^ found that human monocyte‐derived macrophages can phagocytose NETs in a calcium‐dependent manner, and DNase I pretreated NETs can promote their clearance by macrophages. However, this process is not an immune response and does not induce the secretion of inflammatory cytokines. However, LPS‐induced production of IL‐1β, IL‐6, and TNF‐α is enhanced during the phagocytosis of NETs by macrophages. They also found that both recombinant and endogenous C1q can modulate NETs and promote their clearance. Although the study of macrophage phagocytosis of NETs is more comprehensive, the functional status of macrophages and their involvement in the pathogenesis of RA are not yet fully understood.

#### 6.5.7 The relationship of environmental factors with NETs in RA

Smoking has long been recognized as an independent risk factor for RA, and the increased risk of RA associated with smoking may be due to its antiestrogenic effects. Recent research by Lee et al. found that nicotine is an effective inducer of NETs and may exacerbate arthritis in a collagen‐induced mouse arthritis model, leading to harmful effects on RA patients, including those using electronic cigarettes.^17^ Makrygiannakis et al. proposed that tobacco smoke enhances PAD expression in the lungs, leading to the generation of citrullinated proteins, and this may also enhance the induction of NETs.^291^ Similarly, periodontal disease has been identified as a risk factor for RA,^292^ mainly due to infection with Porphyromonas gingivalis, the only known prokaryotic microorganism that expresses PAD homologs. Moreover, immunization with P. gingivalis can induce autoimmune responses to self‐antigens in mice expressing α‐enolase and DR4‐IE.^293^ Vitkov et al. found an increase in NET formation in gingival crevicular fluid in patients with periodontal disease, and P. gingivalis is capable of inducing NET formation,^294^ suggesting that in patients with periodontal disease induced by P. gingivalis, increased NET formation and citrullination induction may promote the generation of citrullinated self‐antigens and AAbs, initiating further enhanced NET formation and autoimmune reactions.^295^

#### 6.5.8 Advancements in the treatment of NETs in RA

It has been observed that the PADs inhibitor Cl‐amidine is effective in a collagen‐induced arthritis model because it reduces IL‐17A‐induced NET formation in neutrophils.^296^ Su et al. found that Tripterygium wilfordii can inhibit neutrophil oxidative burst activity and the formation of NETs induced by inflammatory stimulation, and they also found that Tripterygium wilfordii red pigment can significantly suppress neutrophil activation and the release of NETs induced by PMA.^297^ Wang et al. found that emodin can alleviate the symptoms of mice with AA and may do so by inhibiting the migration of neutrophils to the inflammatory site, reducing the release of proinflammatory cytokines (IL‐1β and IL‐6), and suppressing the inflammatory response of neutrophils.^298^ Jiang et al. found that Danqi Jiangtang capsule can alleviate the symptoms of mice with AA, possibly by inhibiting the migration of neutrophils to the inflammatory site, reducing the release of proinflammatory cytokines (IL‐1β, IL‐6, iNOS), and reversing the delayed apoptosis of neutrophils.^299^ Sun et al. found that Polygonum cuspidatum can inhibit NET formation by neutrophils and mitigate the inflammatory response and tissue damage in RA.^300^ Lu et al. found that Rheum officinale MII may improve symptoms in a mouse AA model by reducing neutrophil migration to the inflamed joint, decreasing the release of proinflammatory cytokines (IL‐1β and IL‐6), and inhibiting the formation of NETs.^301^, ^302^, ^303^, ^304^ These studies suggest that these natural compounds and herbal formulations can be effective in alleviating RA symptoms by targeting NET formation

### 6.6 Adult‐onset Still's disease

Adult‐onset Still's disease (AOSD) is a rare systemic inflammatory disease characterized by various clinical manifestations, typically presenting with high fever, transient rash, polyarthralgia, and hepatosplenomegaly.^305^, ^306^ For the pathogenesis, a cytokine storm of proinflammatory molecules, including IL‐1β, IL‐6, IL‐18, TNF‐α, and macrophage inhibitory factor, released by innate immune cells (mainly neutrophils and monocytes/macrophages), plays a crucial role in the development of this disease.^307^, ^308^ The inflammatory attacks of AOSD are characterized by neutrophilia, suggesting that neutrophils are the major effector cells in the pathogenesis of AOSD.^309^, ^310^ Hu et al.^311^ found that the levels of circulating free DNA (cfDNA) and NET–DNA complexes were significantly increased in AOSD patients compared with healthy controls, and freshly isolated neutrophils from AOSD patients were prone to spontaneous high‐level NET release. Interestingly, NOX inhibitors and mitochondrial clearance agents abolished the enhanced NET release. Moreover, purified DNA from AOSD NETs activated NLRP3 inflammasomes. NET–DNA from AOSD also exerted potent proinflammatory effects by accelerating the activation of CD68+CD86+ macrophages and increasing the expression of IL‐1β, IL‐6, and TNF‐α. Finally, NETs and plasma mitochondrial DNA (mtDNA) copy numbers were significantly increased in AOSD patients, suggesting the involvement of mtDNA in the activation of NLRP3 and inflammatory macrophages. Ahn et al.^312^ also found that the levels of NET molecules, cfDNA, MPO–DNA, and α‐defensins were associated with several disease activity markers of AOSD. Following corticosteroid treatment in AOSD patients, the levels of cfDNA and α‐defensins significantly decreased. The presence of NE‐positive and MPO‐positive inflammatory cells in the skin and lymph nodes of AOSD patients, with fibrous forms found in the lesions. Serum‐induced neutrophil NETosis was observed in active AOSD patients. NET molecules induced the production of IL‐1β by monocytes, representing a novel mechanism in the pathogenesis of AOSD. Another study^313^ also investigated the mechanisms of stress‐induced inflammatory attacks in patients with familial Mediterranean fever (FMF), where neutrophil activation and release of IL‐1β‐bearing NETs were observed. Previous studies on neutrophils have shown that treatment with sera from AOSD patients containing NET components can induce NETosis in neutrophils.^297^ Furthermore, the results suggest that NET molecules play an important role in the pathogenesis of AOSD and can serve as additional biomarkers for monitoring disease activity.^312^ These data suggest that NETs may contribute to the pathogenesis of AOSD.

### 6.7 Familial Mediterranean fever

FMF is the most common inherited autoinflammatory disease, primarily affecting individuals in the Eastern Mediterranean region. Clinically, it is characterized by neutrophil‐induced serosal inflammation (including synovitis, pleuritis, and pericarditis) leading to recurrent, self‐limiting episodes of fever.^314^, ^315^ FMF is associated with mutations in the MEFV gene encoding pyrin protein, which is highly expressed in neutrophils.^316^ Neutrophils are the major effector cells during acute inflammatory attacks in FMF, leading to neutrophilia and massive infiltration of neutrophils at the sites of inflammation.^317^, ^318^ Mutated pyrin reduces autophagic function by impairing its role as a selective autophagy receptor targeting inflammasome components, resulting in the release of IL‐1β, a key cytokine in the disease.^319^, ^320^, ^321^ Recent experimental evidence suggests that neutrophils release abundant NETs in an autophagy‐dependent manner during FMF inflammation. These NETs transfer biologically active IL‐1β to the extracellular space, amplifying the release of IL‐1β in the first 24 h of acute inflammatory attacks. Concurrently, activity of FMF inflammation is inhibited by limiting NET‐induced IL‐1β via the DNase‐mediated disassembly of NET chromatin scaffolds. As a compensatory response, autophagy in neutrophils is decreased, leading to reduced NET release and protecting them from crisis.^322^ Additionally, the development and DNA damage response regulator 1 (REDD1) acts as a modulator of neutrophil function upstream of pyrin, contributing to the release of NETs and regulation of IL‐1β, thereby becoming a new link in the mechanism underlying FMF attacks.^323^

### 6.8 Psoriasis

Psoriasis is a common chronic inflammatory disease, with an estimated prevalence of 2% in Western countries and less than 0.5% in China.^324^ Typical presentations include well‐demarcated plaques or patches with silver‐white scales. Psoriasis patients often experience persistent skin itching and may have comorbidities such as psoriatic arthritis, metabolic syndrome, type 2 diabetes, and cardiovascular disease.^325^, ^326^ In recent years, significant progress has been made in understanding the pathogenesis of psoriasis. Among these advances, the relationship between NETs and psoriasis has received widespread attention and is considered a potential therapeutic target.

#### 6.8.1 Psoriasis, the immune system, and NETs

Studies have shown that the IL‐23/Th17 cell axis plays a key role in the pathogenesis of psoriasis. Under the influence of underlying triggers, normal skin produces an excess of AMPs that form complexes with host DNA, stimulating pDCs to produce IFN‐Is (IFN‐α and IFN‐β) and initiating the development of psoriasis.^327^, ^328^, ^329^ Sun et al.^330^ reported that protein tyrosine phosphatase SHP2 exacerbates psoriasis‐like skin inflammation in mice through ERK5‐dependent NETosis. Targeting SHP2 inhibition significantly improved psoriasis‐like inflammation in these mice.^330^ NETs can also stimulate pDCs to produce a large amount of IFN‐Is, contributing to the processes of SLE, abdominal aortic aneurysm, and hidradenitis suppurativa, among others.^331^, ^332^ NETs can also lower the activation threshold of T cells, increasing their responsiveness to secondary stimuli.^333^

#### 6.8.2 Involvement of NETs in the development of psoriasis

Previous animal and clinical studies have shown that NETs are involved in the pathogenesis and development of psoriasis. The number of neutrophils undergoing NETosis in the peripheral blood of psoriasis patients was significantly increased compared with the control group. NET levels are also significantly elevated in skin lesions of patients with psoriasis.^334^ Clinical studies have shown that oral dimethyl fumarate (DMF) is safe and effective for the treatment of severe psoriasis. After 16 weeks of treatment, 37.5% of patients achieved a 75% reduction in the Psoriasis Area and Severity Index (PASI 75%) and no recurrence within 2 months after treatment.^335^, ^336^, ^337^, ^338^ Importantly, a study^338^ showed that DMF can reduce ROS production, inhibit neutrophil activation, and decrease NET secretion, suggesting that NETs may be involved in the process of DMF‐mediated psoriasis improvement. However, further research is needed to explore the role of NETs in psoriasis. Taken together, the above clinical studies suggest that NETs are closely related to the pathogenesis of psoriasis. In an imiquimod‐induced psoriasis mouse model, intravenous administration of a CI‐amide compound (which inhibits PAD4 and reduces NET production) for 7 consecutive days resulted in a significant decrease in NET levels in the peripheral blood, a reduced number of infiltrating T cells and neutrophils in the dermis, and significantly reduced scaling and acanthosis of the skin. This suggests that inhibiting NET production with a CI‐amide compound can alleviate psoriasis.^339^ On the other hand, by using DNAse to degrade extracellular DNA and reduce circulating NET levels (NET degradation agents), similar results were also obtained in IMQ‐treated mice, with a significant reduction in psoriasis after degradation of NETs.^339^ The animal experiments mentioned above suggest that NETs play a role in the pathogenesis of psoriasis and are involved in the process of psoriasis resolution.

#### 6.8.3 The role of NETs in the initiation of psoriasis

Previous research^340^ has shown that excessive AMPs (mainly LL37) in skin lesions play a pivotal role in the pathogenesis of psoriasis. IL37 in the skin lesions of psoriasis patients mainly originates from activated keratinocytes and neutrophils. It has been found that IL37 can promote neutrophils to engulf host or bacterial (foreign) RNA and guide RNA into the cells, leading to the release of various cytokines and chemokines, as well as the generation of NETs, which occurs in response to minor skin injury. On the other hand, NETs contain various complexes such as IL37–RNA and IL37–DNA. LL37–RNA can induce massive migration and further secretion of NETs in inactive neutrophils through TLR8/TLR13, establishing a self‐amplifying immune activation loop based on repeated activation of neutrophils.^341^ Additionally, the complex of NE and secretory leukocyte protease inhibitor (SLPI) is present in NETs and colocalizes with pDCs in the psoriatic skin lesions, inducing IFN‐I production by pDCs.^342^ Furthermore, NETs can promote high expression of human beta‐defensin 2 (HBD‐2) in keratinocytes, which can also activate pDCs through DNA binding and participate in the early development of psoriasis.^334^ HBD‐2 is expressed at low levels in normal skin and other erythematous scaleskin diseases, and the level of HBD‐2 in circulating blood serum may serve as a potential biomarker to monitor the therapeutic effect and distinguish the severity of psoriasis.^343^ It is evident that the release of NETs is an important factor in the activation of pDCs and the early development of psoriasis, and the mechanism may involve IL37, NE, SLPI, and HBD‐2.

#### 6.8.4 NETs and the maintenance of psoriasis inflammation

In advanced psoriatic lesions, there is often a large accumulation of neutrophils in the epidermis, even forming typical pathological changes such as Kogoj and Munro microabscesses. Neutrophils infiltrating the epidermis produce NETs that directly act on keratinocytes and participate in the maintenance and amplification of the cutaneous inflammatory environment. NETs can stimulate keratinocytes via TLR4 to release inflammatory mediators such as lipocalin‐2 (LCN2), CXCL8, CXCL1, and IL‐36γ. LCN2, in particular, can influence the activity of neutrophils, such as their activation, migration, and infiltration, and may indirectly contribute to the pathogenesis of psoriasis.^344^, ^345^ In an IMQ‐induced mouse model, treatment with antibodies to neutralize LCN2 led to a decrease in neutrophil and NET levels in the skin.^344^ On the other hand, the activation of TLR4 in keratinocytes by NETs synergistically stimulates IL‐36 receptor (IL‐36R), upregulates the expression of MyD88, TRAF6, and TAK1, downstream molecules in the NF‐κB pathway, and activates the transcription and expression of LCN2. LCN2 recruits neutrophils to migrate to the epidermis, and neutrophils subsequently release NETs, establishing a self‐sustaining inflammatory loop.^339^ Furthermore, NETs can reduce the activation threshold of T cells, increasing their response to secondary stimulation.^333^ Additionally, a study also found the presence of NETs in psoriatic skin lesions.^340^ They suggested that AIM2‐mediated activation of IL‐1β through DNA from NETs could exacerbate the inflammatory progression of psoriasis. Clearly, NETs play a significant role in the maintenance of inflammation in psoriasis.

### 6.9 Inflammatory bowel disease

Inflammatory bowel disease (IBD) is a common chronic, recurrent, nonspecific gastrointestinal inflammatory disease, mainly including CD and ulcerative colitis (UC).^346^ The main pathological changes in IBD are abnormal mucosal immunity and inflammatory reactions characterized by the production of proinflammatory cytokines, ROS, reactive nitrogen species, and activation of inflammatory cells. Neutrophils are the major cells involved in tissue inflammation and damage in IBD. The main mechanisms of neutrophils in IBD involve their migration and accumulation at inflammatory sites, as well as the release of inflammatory mediators and large amounts of ROS.^347^, ^348^

#### 6.9.1 Amplification of inflammatory response in IBD by NETs

IBD is characterized by chronic, recurrent, nonspecific inflammatory reactions in the intestinal mucosa. Studies have found that neutrophil infiltration is correlated with the severity of endoscopic findings and systemic inflammation indices. Serum levels of C‐reactive protein, lactoferrin, and calprotectin in feces can serve as sensitive markers of intestinal inflammation. It is worth noting that lactoferrin and calprotectin are key components of NETs.^349^, ^350^, ^351^, ^352^ Although these molecules can be secreted independently of NETs, current research suggests that they play important roles in the occurrence and development of inflammation in IBD. A proteomic analysis^349^ found elevated levels of 11 proteins related to neutrophils and NETs in UC tissue samples, and this was validated by confocal microscopy through colonic tissue samples of UC patients. Dinallo et al.^353^ found increased expression of PAD4 and CitH3 in colonic tissues of UC patients compared with the control group. In vitro experiments also showed that NETs increased the production of proinflammatory cytokines, such as IL‐1β and TNF‐α, by lamina propria mononuclear cells (LPMCs) of UC patients.^353^ The potential mechanism involved the enhancement of ERK1/2 phosphorylation in LPMCs by NETs, and selective inhibition of ERK1/2 with PD98059 significantly reduced the production of proinflammatory cytokines induced by NETs.^353^ Furthermore, in in vivo experiments, DSS‐induced colitis in BALB/c mice led to an increased expression of PAD4 and CitH3 in colonic tissues, and the use of a selective PAD4 inhibitor, Cl‐amidine, effectively alleviated the inflammatory response induced by DSS.^353^ NETs may amplify the inflammatory response in IBD by acting on cell‐free DNA (cf‐DNA) scaffolds that are bound by proteases, granule proteins, and enhancers of proinflammatory cytokine production in macrophages.^354^, ^355^

#### 6.9.2 NETs aggravate tissue damage in IBD

Damage to intestinal epithelial cells (IECs) leading to impaired gut barrier function is one of the pathological features of UC. Damage to ECs can also result in delayed healing of local ulcers and exacerbate intestinal injury.^126^ Marin‐Esteban et al. demonstrated that NETs induced by Escherichia coli were able to disrupt F‐actin cytoskeletal structure of human intestinal epithelial Caco‐2/TC7‐like cells. This may be due to the damaging effects mediated by histones, proteases, and ROS, which are embedded in the scaffolding of free DNA.^356^, ^357^ These proteins can be used as biomarkers for the diagnosis and prognosis of IBD. These molecules are released during the formation of NETs and are likely to be important in the occurrence and development of IBD.^358^ Dinallo et al.^353^ found that the expression of PAD4 and CitH3 was increased in colonic tissues of UC patients compared with the control group. In vitro experiments also showed that NETs enhanced the production of proinflammatory cytokines, such as IL‐1β and TNF‐α, by LPMCs from UC patients.^353^ The possible mechanism involved enhanced phosphorylation of ERK1/2 in LPMCs induced by NETs, while selective inhibition of ERK1/2 with PD98059 significantly reduced the production of proinflammatory cytokines induced by NETs. In in vivo experiments, DSS‐induced colitis in BALB/c mice resulted in a significantly higher expression of PAD4 and CitH3 in colonic tissues compared with the control group. Treatment with the selective PAD4 inhibitor, Cl‐amidine, effectively alleviated the inflammatory response induced by DSS. Additionally, NETs can induce macrophages to release proinflammatory cytokines.^353^ Recent studies showed that NETs significantly enhanced the response of monocytes derived macrophages to low‐dose LPS, leading to increased release of TNF‐α, IL‐6, and monocyte chemotactic protein‐1 (MCP‐1), while treatment with DNase significantly reduced the activation of macrophages and the secretion of TNF‐α, IL‐6, and MCP‐1.^354^, ^355^ Therefore, NETs may amplify the inflammatory response in IBD by acting on cf‐DNA scaffolds that are bound by proteases, granule proteins, and enhancers of proinflammatory cytokine production in macrophages.

#### 6.9.3 NETs exacerbate tissue damage in IBD

IEC injury leading to impaired intestinal epithelial barrier function is one of the pathophysiological manifestations of UC. Additionally, EC damage can delay local ulcer mucosal healing and worsen intestinal injury. The study by Gottlieb et al.^126^ revealed that the expression of NETs is significantly higher in both pediatric UC and CD patients compared with healthy controls. Marin‐Esteban et al.^356^ demonstrated that NETs generated by differentiated PLB‐985 cells, a neutrophil cell line, induced by Escherichia coli, can disrupt the F‐actin cytoskeleton of human intestinal‐like Caco‐2/TC7 cells. This effect may be attributed to the damaging effects of embedded histones, proteases, and ROS on the free DNA scaffold. Additionally, some studies have suggested that neutralizing antibodies to ANCAs, including neutrophil proteins such as PR3, MPO, NE, histones, and membrane proteinase G, which are important components of NETs, can serve as biomarkers for the diagnosis and prognosis of IBD.^357^ These molecules are released during NET formation, which is likely a significant factor in the production of ANCAs. Furthermore, in vivo experiments have shown that ANCA can induce NET formation in neutrophils and activate complement, leading to endothelial damage. Dendritic cells exposed to NETs can induce the production of ANCA, and ANCA against MPO can be detected in the serum of IBD patients. Interestingly, compared with CD, ANCA against PR3 is easier to detect in tissue specimens from patients with UC, allowing for differentiation between these two diseases.^358^ The potential reason for this difference is the varying composition of NETs released in these two conditions. Additionally, as an important component of NETs, the level of NE is elevated in the feces of IBD patients, contributing to the disruption of the epithelial barrier function. Treatment with an elastase inhibitor can improve experimental colitis in mice. On the other hand, recent studies have reported that NETs mediate vascular endothelial dysfunction in small‐vessel vasculitis and sepsis through histamine and PR3, which has been validated in an in vitro coculture system of neutrophils and ECs.^359^ Furthermore, apart from causing endothelial damage and promoting intestinal inflammation and ulcers, damaged ECs can transform into procoagulant phenotypes, potentially in collaboration with activated platelets, leading to the formation of extraintestinal thrombi. Notably, treatment with DNase significantly reduces the death of both IECs and ECs in DSS‐induced colitis in mice.^355^ Furthermore, there is abundant expression of NETs in the body of IBD patients, and their expression levels are positively correlated with disease activity. Patients in the active phase of UC have a higher expression of NETs compared with patients with active CD. Animal models have shown that NETs are significantly expressed during the acute phase of colitis and decrease with disease remission. Inhibiting the expression of NETs can alleviate disease activity, inhibit tissue cell apoptosis, improve epithelial barrier function, and suppress the expression of inflammatory factors. NETs can damage the intestinal epithelial barrier function, which is regulated through the PI3K/Akt pathway. IGF‐1 can synergistically regulate Akt phosphorylation to improve the impact of NETs on intestinal mucosal barrier function. Therefore, NETs may promote the occurrence and development of IBD by disrupting the intestinal mucosal epithelial barrier function and damaging ECs.

#### 6.9.4 NETs promote hypercoagulability in IBD

Venous and arterial thromboembolism is a major cause of morbidity and mortality in IBD patients. HE et al.^360^ found that NETs promoted hypercoagulability in IBD patients and increased the occurrence of thromboembolic events. TNF‐α is increased in the peripheral blood of IBD patients, leading to the release of NETs. The above processes are mainly related to the adhesion of platelets to neutrophils and the interaction between host cells and microorganisms. NETs induced ANCAs can cause phosphatidylserine (PS) externalization and provide binding sites for the complex of coagulation factors FXa and prothrombinase, further promoting thrombin and fibrinogen formation. Thrombin and FXa, in addition to being passive mediators of coagulation, also participate in the aggravated and prolonged inflammation in IBD patients and mouse models through PAR signaling.^361^ Thus, NETs, PS, and the induced thrombin and FXa play a bridging role between the immune response, inflammation, and thrombosis in IBD. Additionally, NETs are involved in thrombus formation, usually by binding to TLR2 and TLR4 or by binding to the receptor for IgG complexes.^362^ Furthermore, in vitro studies have shown that TLR2 and TLR4 antagonists can reduce the percentage of PS‐exposing platelets and the level of PS‐exposing platelet microparticles. Moreover, TLR4 antagonist can reduce the expression of PS in human umbilical vein ECs.^362^ Therefore, NETs may play an important role in the regulation of mucosal immune responses, restoration of gut microbiota imbalance, and repair of damaged intestinal epithelial barriers.

#### 6.9.5 The relationship between NETs and IBD treatment

Various drugs targeting NETs have been applied clinically, such as in cystic fibrosis, gout, and SLE. These drugs are expected to play a role in the treatment of IBD.^363^ Currently, conventional treatments for IBD (including aminosalicylates, steroids, etc.) have some efficacy, but some patients have poor responses to standard‐dose steroids, while others have long‐term steroid resistance or dependence. Biologics, represented by anti‐TNF‐α agents, have greatly improved the prognosis and survival of IBD patients.^364^ It has been found that TNF‐α can induce neutrophils of UC patients to release NETs, and the anti‐TNF‐α antibody infliximab (IFX) significantly reduced the expression of PAD4 and NETs in colon tissues of UC patients, providing support for the clinical treatment of refractory IBD with anti‐TNF‐α agents.^353^ Furthermore, animal experiments have showed that when antibiotics or mice were reset to a germ‐free environment, the UC symptoms were significantly relieved, demonstrating that gut microbiota plays a role in the pathogenesis of UC.^365^ Vong et al.^366^ mentioned that treatment with antibiotics or exposure to a sterile environment significantly reduced the expression of PAD4 and CitH3 in colonic tissues of DSS‐treated mice. Additionally, probiotics such as Lactobacillus rhamnosus could inhibit the production of ROS and NETs. This phenomenon was observed in in vitro experiments using bone marrow neutrophils from mice and the human leukemic cell line HL‐60.^367^ Therefore, probiotics may regulate mucosal immune responses through NETs, improve the imbalanced gut microbiota, and repair damaged intestinal mucosa, thus serving as adjunctive therapy for IBD. In addition, due to the complexity of NETs formation and the diversity of their components, several substances can directly or indirectly act on NETs and may become precursors for the treatment of IBD, such as DNase 1 acting on cf‐DNA, selective PAD4 inhibitors like Cl‐amidine, ROS scavengers like N‐acetylcysteine, MPO inhibitors like PF1355, and endogenous NETosis inhibitors like prostaglandin E2.^353^, ^355^, ^363^ Therefore, NETs‐targeted therapy will provide more treatment options for IBD. In summary, since the discovery of NETs more than a decade ago, research on NETs has become a hot topic in the study of various diseases. Due to the presence of autoantigenic substances in the composition of NETs themselves, NETs are closely related to the occurrence and development of autoimmune/inflammatory diseases. IBD is a type of autoimmune and inflammatory disease affecting the intestines. Research has found that NETs may play an important role in IBD by amplifying inflammatory responses, aggravating tissue damage, and promoting hypercoagulability. NETs‐related molecules are likely to become targets for IBD treatment and intervention. However, the specific mechanisms of NETs in IBD and whether there are differences in the roles of NETs in UC and CD are not yet clear. Therefore, further high‐quality in vitro and in vivo research is needed to provide new insights for the development of treatment strategies and specific targeted drugs for IBD.

### 6.10 NETs in the pathogenesis of multiple sclerosis

Multiple sclerosis (MS) is the most common immune‐mediated inflammatory disease affecting the CNS, with an estimated 2.5 million people worldwide affected.^368^ The pathological characteristics of MS are inflammation, demyelination, neurotoxicity to neurons, and the development of multiple chronic inflammatory CNS lesions, with no clear cause or treatment.^369^

Neutrophils, in particular, have been implicated in the development and pathophysiology of MS. It is suggested that neutrophils primarily contribute to the onset of MS by affecting the periphery rather than the CNS.^370^ Neutrophils exhibit multiple functions that influence the mechanisms of MS pathogenesis,^371^ including (1) release of inflammatory mediators and enzymes such as IL‐1β, MPO, and various proteases, (2) destruction and phagocytosis of myelin phospholipids as fragments, (3) release of NETs, (4) production of ROS, (5) disruption of the blood–brain barrier (BBB), and (6) generation and presentation of autoantigens. Among these, NET release is a newly discovered function that mediates the pathogenesis of MS,^372^ and one key question is whether neutrophils exhibit predominantly proinflammatory functions or if they are also involved in the resolution of chronic inflammatory responses in MS. Studies have shown that circulating NETs are increased in MS patients compared with healthy controls, which may be due to the initiation of neutrophil activation in a chronic inflammatory environment.^373^ Elevated levels of circulating NETs have been found in a subset of RRMS patients, with a significant increase observed in male patients and worse prognosis in comparison with female patients.^374^, ^375^ This may indicate gender‐dependent molecular differences in the disease mechanism, as emphasized by transcriptomic studies.^376^ Recent findings in MS suggest that NETs may exert cytotoxic effects on the BBB and induce damage to neighboring neurons and other CNS cells.^375^ This is supported by studies in experimental autoimmune encephalomyelitis (EAE), which show that the consumption of NET‐associated proteins such as MPO and NE reduces disease severity and increases BBB integrity.^376^, ^377^ Allen et al.^378^ demonstrated that neutrophils in mice induce a proinflammatory, neurotoxic phenotype through migration and activation of brain vascular endothelium, which subsequently leads to NET release. However, evidence of NETs contributing to BBB breakdown in MS still needs to be established. As suggested by Paryzhak et al., another role of NETs is the cleavage of circulating ICs in MS.^379^ They highlight the potential of NET‐associated proteases to modify the glycans of internal epitopes. Recent evidence shows that NETs can activate Th17 cells, recruiting neutrophilic cytokines such as IL‐17, and the interaction between neutrophils and IL‐17 significantly delays the onset and reduces the severity of EAE.^380^ Moreover, the severity of EAE is reduced and BBB integrity is restored by lowering NET‐associated proteins such as MPO and NE.^381^ These studies elucidate the relevance of NET release in the pathogenesis of MS; however, the functional connection between these two phenomena remains elusive.^382^

In terms of intervention, studies have shown that CXCR2 antagonists effectively delay and alleviate clinical manifestations and infiltration of inflammatory cells in the CNS in mice; relieving the disease by preventing neutrophil migration across the BBB and inhibiting NET formation at the site of inflammation, thereby reducing the destruction of the BBB.^383^ Fingolimod (FTY720), an immune modulator, has been approved for the treatment of MS, and recent research^384^ suggests that FTY720 induces NET release by a NOX‐independent pathway. However, further studies are needed to establish the functional interplay between NETs and FTY720.^384^ NETs have been reported to enhance autoreactive T‐cell responses and drive the pathogenesis of MS. Targeting the formation of NETs or their components may be a potential therapeutic strategy. In conclusion, MS is a complex immune‐mediated inflammatory disease with a wide range of genetic and environmental factors involved. Neutrophils, particularly their release of NETs, play a role in the onset and pathogenesis of MS. These findings contribute to a better understanding of the disease mechanisms in MS and may provide new insights for the development of targeted therapies. Further research is needed to fully elucidate the functional role of NETs in MS and their potential as therapeutic targets.

### 6.11 Antiphospholipid syndrome

#### 6.11.1 NETs promote thrombus formation in APS

APS is a rare and complex autoimmune disease characterized by recurrent venous and/or arterial thrombosis and pregnancy complications in the presence of antiphospholipid antibodies (aPL), including anticardiolipin antibodies and anti‐β2 glycoprotein I (anti‐β2GPI) antibodies, as well as lupus anticoagulant.^385^ Recent studies have shown that NETs play a significant role in thrombus formation in APS.^386^ Mauracher et al.^386^ found differences in neutrophil subpopulations, their activation potential, and NET formation between high‐density neutrophils and LDNs in APS patients and control groups. Both LDN subpopulations exhibited higher baseline activation and lower responsiveness to stimulation, but showed higher NET formation.^386^ Jonatan et al. found a reduced capacity to degrade NETs in subsets of APS patients, which correlated with specific clinical manifestations and antibodies targeting NET components. Thrombotic APS was associated with increased NET formation, particularly in patients with poor prognosis, such as triple‐positivity for aPL and recurrent thrombosis.^387^ Neutrophils contribute to thrombus formation in APS by mechanisms that involve circulating neutrophils being triggered by aPL to accelerate thrombus formation, with NETs playing an in vivo role in aPL‐mediated venous thrombosis.^388^ Foret et al.^389^ found that NETosis, induced by anti‐β2GPI antibodies, exacerbated the prothrombotic phenotype in APS, especially in high‐risk patients. Targeting NETs in these high‐risk patients reduced antiprothrombinase resistance and offered a novel therapeutic approach for APS, in addition to antithrombotic treatment.^389^ The release of NETs mediated by ROS and TLR4 signaling induced by the interaction between neutrophils and anti‐β2GPI antibodies has also been implicated in APS pathogenesis.^390^ Experimental data show that neutrophil depletion, TLR‐4 mutation, deoxyribonuclease (DNase) administration, Mac‐1 antagonism, P‐selectin glycoprotein ligand‐1 inhibition, and adenosine A2a receptor stimulation all reduce thrombosis in aPL‐treated mice.^391^

In APS, aPL contacts neutrophil surfaces, bypassing normal homeostatic mechanisms, and directly triggers NET release.^392^ In fact, even when evaluated between thrombotic events, circulating levels of NETs are higher in APS patients compared with healthy controls, and neutrophils from APS patients exhibit lower thresholds for spontaneous NETosis in ex vivo culture.^392^ Additionally, neutrophils from APS patients appear to have increased adhesive potential, which depends on the activation form of the integrin Mac‐1, leading to enhanced NET release.^393^ In a mouse model of aPL‐mediated venous thrombosis, neutrophil depletion, disruption of neutrophil–endothelial interactions, and digestion of NETs were all found to be protective.^394^ Mac‐1‐mediated augmented adhesion of neutrophils in APS patients, which could lower the threshold for neutrophil–endothelial interactions, NETosis, and possible thrombotic events.^395^ Lu et al.^396^ found that anti‐ApoA‐I‐ associated high‐density lipoprotein (HDL) and low‐density HDL from APS patients exhibited higher levels of neutrophil adhesion and increased ROS production, both of which were linked to NET formation. It has been reported that adenosine receptor agonists can prevent NETosis and thrombus formation in APS.^397^ Defibrotide increased intracellular cyclic AMP levels in neutrophils and attenuated its inhibitory effect on NET formation by blocking adenosine A2A receptors or inhibiting cyclic AMP‐dependent protein kinase A.^398^ The intersection of NETs, complement, and coagulation is a growing area of research.^399^ NETs can directly activate complement, thereby promoting blood coagulation through their effects on tissue factors and platelets. Conversely, activated complement can promote NET formation, and NETs themselves can act as scaffolds for thrombus formation.^399^ It has been reported that active SLE patients with reduced C3 and C4 complement levels typically have low levels of degradation of NETs, indicating complement activation.^400^ Zuo et al.^401^ found a significant correlation between elevated anti‐NET IgM levels and reduced complement C4 levels in APS, suggesting a potential association of anti‐NET activity and complement activation. As reported by van et al., NET formation is associated with the occurrence of antinuclear antibodies in SLE and APS.^402^ Anti‐NET antibodies may play a coordinating role in the complex relationship between NETs, complement, and coagulation in thromboinflammatory diseases. Further research seems necessary to elucidate the mechanistic connections between anti‐NET activity and complement activation.

In summary, these studies suggest that there is excessive NET formation and impaired NET degradation in APS, and both mechanisms may amplify the impact of NETs on thrombus formation. However, many questions remain, including the extent to which NETs contribute to the obstetric and noncriteria manifestations of APS. One study showed that coenzyme Q10 improved NET extrusion and downregulated oxidative stress, intracellular elastase, and MPO in neutrophils; improved endothelial function, decreased expression of prothrombotic and proinflammatory mediators in monocytes, inhibited phosphorylation of thrombosis‐related protein kinases, and reduced oxidative stress and depolarized mitochondria in monocytes percentage. In patients, the use of the antioxidant coenzyme Q10 has been suggested as a supplementary strategy to inhibit NETs.^403^ In addition to these APS‐targeted studies, several mouse studies have shown that regulation of NETs has a protective effect on thrombus formation and pregnancy rates. Methods used include TLR4 mutations, administration of DNase, PAD inhibitors, and Janus kinase pathway inhibitors.^404^ Due to their relevance to clinical practice, drugs commonly prescribed to APS patients such as aspirin, heparin, antimalarials, and vitamin D have been shown to inhibit NETosis in certain cases.^405^ Therefore, targeting NETs may hold promise as a therapeutic approach for PAPS and SLE.

### 6.12 ANCA‐associated systemic vasculitis

ANCA‐associated systemic vasculitis (AASV) is a systemic autoimmune disease caused by the interaction of multiple factors, including genetics, environment, and immune dysregulation. It mainly includes Wegener's granulomatosis, MPA, Churg‐Strauss syndrome, and ANCA‐associated crescentic glomerulonephritis. Neutrophils play a crucial role in the pathogenesis of AASV.^406^, ^407^ ANCA is a type of AAb targeting the primary granule components of neutrophils and monocyte lysosomes. It is a characteristic serological marker of AASV. The main target antigens of ANCA are PR3 and MPO, which are expressed and released by activated neutrophils. The expression and secretion of PR3 may also be present on the surface of ECs.^408^, ^409^ ANCA activates neutrophils and damages tissues through interaction with target antigens on cell surfaces, contributing to local vascular inflammation.^410^ Studies have shown that NETs are one of the sources of autoantigens in AAV, and dysregulated NET regulation can induce the production of AAbs and exacerbate the severity of AAV.^411^

#### 6.12.1 The role of NETs in the pathogenesis of AAV

**FIGURE 8:**
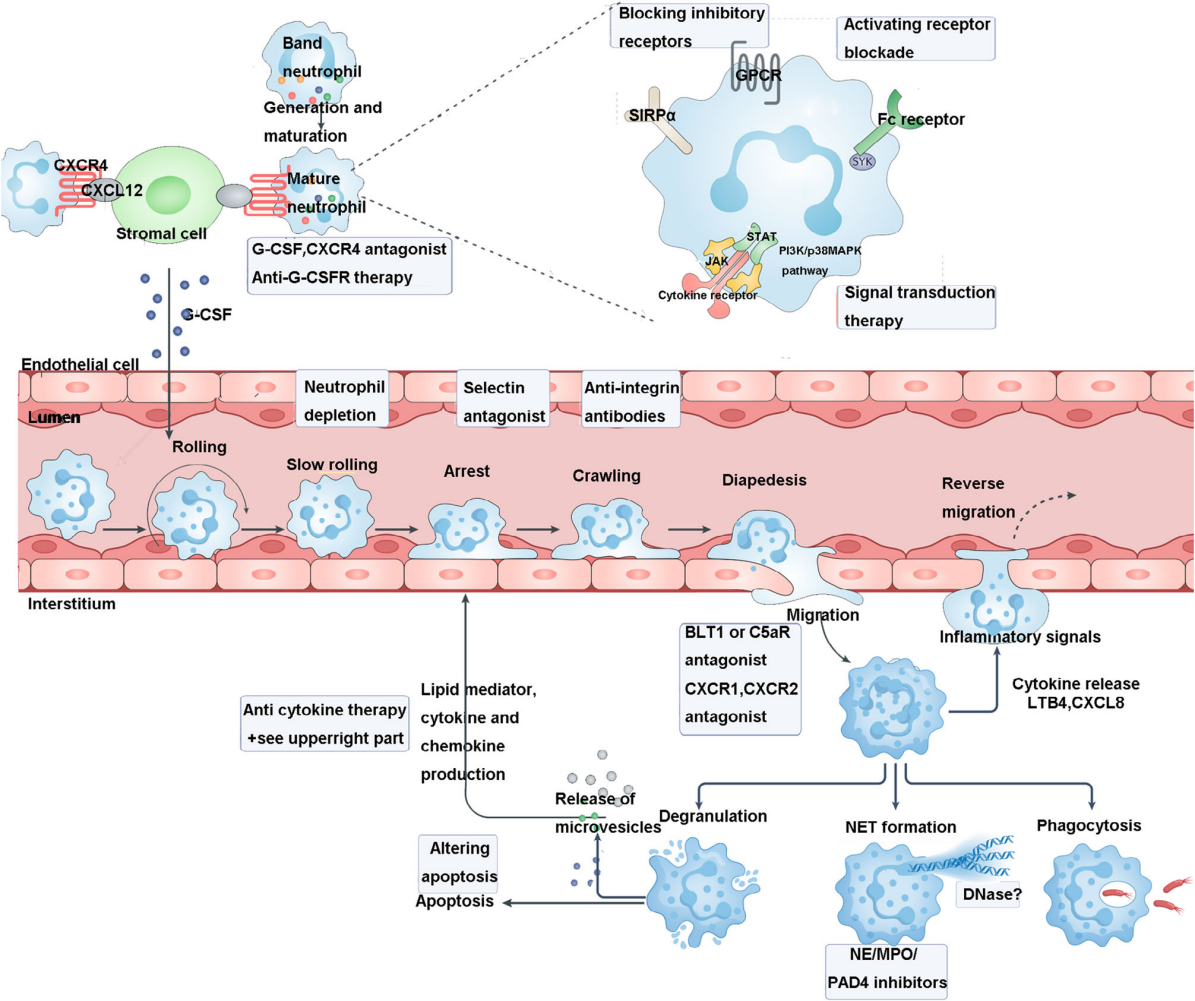
The role of NETs in the pathogenesis of AAV. (Strategies to suppress neutrophil activation and recruitment include targeting cytokines (e.g., IL‐1, IL‐8) and receptors (e.g., C5a receptor) to reduce immunosuppressive neutrophils, modulating neutrophil‐derived products, and targeting neutrophil extracellular traps (NETs) by inhibiting formation, activating degradation, or neutralizing components like MPO and elastase. Created by BioRender, with reference to Ref. ^2^.)

Neutrophils undergo cell lysis and release NETs after the exposure of self‐antigens on their surfaces and interaction with activated/damaged ECs.^412^ ANCA can induce neutrophil degranulation, release ROS, and trigger the formation of NETs through cell factors such as TNF‐α, LPSs, and complement factor 5a.^413^, ^414^ These cell factors induce the expression of endothelial selectins, facilitating the migration of neutrophils in the blood vessel and intercellular spaces.^415^ They also increase the expression of self‐antigens on the surface of neutrophils, such as MPO, PR3, and other neutrophil granule proteins. Consequently, ANCA forms soluble ICs with self‐antigens, and the Fc portion of AAbs binds to FcgRs, ultimately leading to neutrophil activation, release of MPO, NE, and PR3, and the disintegration and extrusion of chromatin from the nucleus, resulting in the formation of NETs, leading to neutrophil death. This subsequently causes small vessel necrotizing inflammation, EC death, increased vascular permeability, and fibrin deposition, followed by the recruitment of monocytes and macrophages, ultimately leading to granuloma formation.^416^ Excessive exposure to NETs can damage ECs, leading to vascular pathology and contributing to the development of AAV. Conversely, ANCA and other humoral factors, such as damage‐associated molecular patterns (DAMPs), further induce the formation of NETs, forming a vicious cycle in the pathogenesis of AAV.^417^ (1) High mobility group protein 1 (HMGB1) has been shown to be involved in ANCA‐induced NET formation.^418^ HMGB1 activates neutrophils by increasing the translocation of ANCA antigens, leading to the activation of NETs and promoting ANCA‐mediated respiratory burst and degranulation.^419^ Studies have shown that HMGB1 increases NET release through TLR2, TLR4, and RAGE pathways in a nicotinamide adenine dinucleotide phosphate (NADPH) oxidase‐dependent manner.^419^ (2) Tang et al. demonstrated that ANCA induced autophagy in neutrophils through lysosome‐associated membrane protein‐2, promoting NETosis and exacerbating vascular injury.^420^ In summary, overwhelming exposure to exogenous antigens (such as drugs or microbes) and self‐antigens (due to mutations or intracellular components) leads to the conversion of nonpathogenic natural ANCA to pathogenic ANCA under the influence of genetic and environmental factors. This leads to neutrophil lytic death and the formation of NETs, which cause vascular injury. NETs can activate ANCAs by disrupting immune tolerance, leading to the development of AAV. Thus, ANCA‐induced NETosis damages ECs, causing vascular inflammation, while excessive NET activation induces ANCA production, creating a vicious cycle. The interaction of ANCA with NETs is reciprocal and mutually causal, representing two important factors in the occurrence and development of AAV (Figure 8).

#### 6.12.2 Treating AAV by targeting NETs

Currently, there are drugs targeting NETs being studied in animal models and in vitro experiments for AAV.^421^ CI‐amidine, a PAD4 inhibitor, has been identified as a new target drug.^422^ Rao et al.^423^ demonstrated that PAD inhibitors can reduce NET formation, improve endothelial function, decrease serum MPO–ANCA levels, and inhibit vascular inflammation in an MPO–ANCA mouse model. Alvelestat and Bay85‐8501 are two drugs targeting NE. Alvelestat, a third‐generation NE inhibitor, has been shown to effectively inhibit NETosis and reduce inflammation in acute lung injury/acute respiratory distress syndrome mouse models.^424^, ^425^ Bay85‐8501, a fifth‐generation NE inhibitor, exhibits greater safety and efficacy compared with previous inhibitors. It has been observed to significantly reduce NET formation in in vitro experiments.^424^ Both drugs have shown good safety and tolerability in clinical trials for the treatment of respiratory diseases and have become candidate drugs to block NETs formation in AAV. In an MPO–AAV mouse model, treatment with Alvelestat and Bay 85–8501 successfully reduced NET accumulation in glomeruli and MPO deposition, resulting in reduced glomerular injury and decreased clustering of CD4 T cells, CD8 T cells, macrophages, and neutrophils in glomeruli, thus inhibiting the progression of MPA and renal damage. Moreover, Bay85‐8501 treatment significantly reduced ANCA titers in mice.^424^, ^426^ Phosphatidylinositol 3‐kinase γ (PI3K‐γ) enhances T‐cell activity and increases IL‐17 levels, contributing to ANCA autoimmune responses.^427^ Genetic ablation of PI3K‐γ reduces NETosis and ANCA production. Treatment with a PI3K‐γ‐specific inhibitor in an MPA mouse model resulted in reduced ANCA titers and severity of renal and lung injury, indicating that blocking PI3K‐γ to inhibit NETosis effectively improves MPA and prevents kidney and lung damage.^427^ Kimura et al.^428^ revealed that PI3K‐γ blockade can inhibit NETosis, subsequently preventing the production of ANCA and subsequent kidney and lung damage. PI3K‐γ inhibitors have already shown efficacy in improving symptoms in animal models of RA, and PI3K‐γ/δ dual inhibitors have entered Phase I‐III clinical trials for the treatment of cancer and asthma.^429^ Schreiber et al.^430^ reported that ANCA induces NETosis through receptor‐interacting serine/threonine‐protein kinase 1/3 (RIPK1/3) and mixed‐lineage kinase domain‐like (MLKL) dependent necroptosis. Currently, RIPK1 inhibitors are in Phase II clinical trials for the treatment of IBD, psoriasis, and RA.^430^ Thus, inhibiting PAD4, NE, PI3K‐γ, and RIPK1 can effectively reduce NET production in AAV patients, offering potential targets for AAV treatment. The main treatment options for NETs degradation in the body are DNase enzymes; however, in autoimmune patients, there is a widespread phenomenon of decreased levels or activity of DNase, resulting in the persistence of NETs.^431^ Adeno‐associated virus vectors (AAVec) can deliver DNase I continuously, providing a one‐time treatment for AAV. AAVec technology has been successfully used in the treatment of human blindness, coagulation disorders, and neuromuscular diseases.^432^ Therefore, AAVec technology may be applicable for the treatment of AAV.

In conclusion, the generation and degradation of NETs play important roles in the pathogenesis of AAV. Targeting NETs activation or promoting their degradation provides new directions for the treatment of AAV.^433^, ^434^ Studies have validated the role of NETs in AAV through clinical samples and animal experiments, and research into NETs as a therapeutic target is also being extensively conducted.^421^, ^435^ Further exploration of the molecular signaling pathways related to NETosis and NETs involvement in ANCA formation is of significant importance for understanding the pathogenesis of autoimmune diseases and discovering new clinical treatments. More basic research and clinical trials are needed in the future to provide new insights for AAV treatment strategies and the development of specific targeted drugs, to offer more personalized guidance for disease management.

### 6.13 Type 1 diabetes mellitus (T1DM)

T1DM is the main type of diabetes in children, accounting for about 80%. However, in reality, T1DM can occur at any age.^436^ Studies have shown that up to 50% of patients develop T1DM during adulthood, with approximately half of them being misdiagnosed as type 2 diabetes.^437^, ^438^ Wang et al.^439^ found that the levels of elastase and PR3 in the serum of T1DM patients were elevated and positively correlated with the degree of beta cell autoimmunity, indicating a close relationship between NETs and the occurrence and development of T1DM.^439^ Diana et al.^440^ found that pancreatic neutrophils and beta‐1a cells cooperate to activate pDCs and initiate the development of T1DM. Additionally, NETs were found to be present in pancreatic tissue of T1DM patients.^440^ You et al.^441^ found that NET formation, particularly the changes in serum PAD4 levels, were closely associated with intestinal permeability in T1D but not T2D patients, suggesting that microbial invasion and the release of neutrophil cytoplasmic antigens during NET formation may be important pathogenic factors in T1D.

### 6.14 Bullous pemphigoid

Bullous pemphigoid (BP) is an acquired autoimmune blistering disease characterized by tense blisters predominantly on the trunk and extremities. Circulating AAbs in BP patients, such as anti‐BP180 and anti‐BP230 antibodies, target components of the hemidesmosomal complex at the dermo‐epidermal junction, including BP180 (BPAG2 or type XVII collagen) and BP230 (BPAG1e). These antibodies activate complement, induce degranulation of mast cells, and recruit neutrophils and eosinophils, leading to the release of various proteases that degrade the normal tissue architecture at the dermo‐epidermal junction, resulting in blister formation.^442^ As early as 1997, Liu et al.^443^ found that injection of antimouse BP180 antibodies in a passive transfer mouse model induces blister formation, while depletion of neutrophils using antineutrophil‐specific antibodies in mice leads to IgG and C3 deposition at the dermo‐epidermal junction without inflammation or blister formation. This confirms the crucial role of neutrophils in the development of BP, where the presence of neutrophils determines whether blisters form. Oswald et al.^444^ demonstrated in animal experiments that specific monoclonal antibodies against Ly‐6G can significantly reduce neutrophil counts in peripheral blood of BP animal models. Predepleting neutrophils using Ly‐6G monoclonal antibodies resulted in a significant reduction in clinical activity scores of blister formation in the BP animal model, indicating that neutrophil depletion can partially inhibit blister formation. Studies have shown that reducing neutrophil accumulation by 30% is sufficient to inhibit subepidermal blister formation; thus, the quantity of neutrophils directly correlates with disease severity.^445^ Lin et al.^446^ found that mice lacking NE failed to develop BP. The main role of NE is the degradation of the extracellular domain of BP180, resulting in the formation of peptides P561 and P506. Local injection of NE in B6 mice promotes neutrophil infiltration into the skin, while injection of alpha‐1 proteinase inhibitor completely blocks neutrophil chemotaxis. This is mainly because the peptide products of NE degradation of BP180, such as P561, possess chemotactic properties for neutrophils. In addition to cleaving BP180 and extracellular matrix, NE and MMP‐9 participate in promoting the aggregation of late‐stage neutrophils.^443^ Moreover, neutrophils and monocytes can produce CXC chemokine ligand 10 (CXCL10), which activates extracellular regulated kinase 1/2, P38, and PI3K (Akt) signaling pathways,^447^ further activating nicotinamide‐adenine dinucleotide phosphate oxidase (NADPHO) to generate ROS.^448^ ROS, along with the fibrinolytic system, can activate the precursor of MMP‐9, leading to its production. Neutrophils and mast cells produce IL‐17, promoting the production of CXCL10, and further upregulating the production of MMP‐9 and NE.^449^

### 6.15 Systemic sclerosis

Systemic sclerosis (SSc) is a rare connective tissue disease characterized by skin and organ fibrosis, vascular abnormalities, and autoimmune features. Based on the extent of skin fibrosis, SSc can be divided into two clinical subsets: limited cutaneous SSc (lcSSc) and diffuse cutaneous SSc (dcSSc).^450^ In addition to skin involvement, 20–40% of SSc patients develop interstitial lung disease (ILD), which can lead to respiratory failure and death.^451^ The disease is characterized by endothelial dysfunction, fibroblast dysfunction, and immune cell dysfunction, resulting in heterogeneous organ damage that compromises patient survival. Free radicals and ROS play important roles in the pathophysiology of SSc. Current research has shown that oxidative stress is involved in the pathogenesis of SSc, and it is known that polymorphonuclear neutrophils (PMNs) produce large amounts of ROS in the disease process.^452^ In addition, MPO–DNA complexes and citrullinated H4 histone have been found in the plasma of SSc patients, suggesting the presence of dysregulated NETosis in this disease.^453^ Other studies have also confirmed the occurrence of NETosis in SSc patients.^454^ Neutrophils in SSc patients exhibit functional defects that affect cell migration, NET formation, and bacterial phagocytosis.^454^ In this study, the authors observed that the concentration of MPO–DNA complexes in the plasma of early and active SSc patients was significantly higher compared with that in late‐stage SSc patients during capillaroscopy examination. Additionally, DNA fibers were observed in skin biopsies of SSc patients. These potential NET structures were found to be modified by CXCL4, and the CXCL4–DNA complexes may be involved in immune activation, particularly in the production of IFN‐I in early active SSc patients.^455^ Didier et al.^456^ demonstrated that both serum factors and PMN activation state contribute to enhanced production of NETs in vitro. However, they found that the type of AAbs does not seem to affect the level of NET formation in SSc, although the role of AAb in SSc‐related NETosis cannot be ruled out.^456^ Furthermore, ICs containing ribonucleoproteins frequently detected in SSc serum have been shown to induce NETosis in SLE patients, representing interesting serum candidates for SSc‐related NETosis.^457^ Microparticles expressing DAMPs released from activated platelets seem to activate PMNs and may also contribute to NETosis in SSc serum.^467^ Abnormalities in the PMN pathway may support abnormal initiation of NETosis favoring NETosis in SSc patients. In fact, the levels of activated ERK, p38, and JNK in SSc PMNs were found to be much higher compared with control cells. It is worth noting that ERK is known to be involved in the production of NETs.^458^, ^459^ Additionally, IL‐8 has been found to be a key cytokine in PMN‐induced NETosis and is increased in the serum of SSc patients.^460^

Latest research demonstrates that plasma samples from SSc patients with high levels of fMet induce activation of neutrophils through an FPR1‐dependent mechanism.^461^ Mitochondrial component fMet plays an important role in promoting neutrophil‐mediated inflammation (excessive neutrophil activation and formation of NETs, NETs) in SSc. In a recent study,^462^ a significant amount of microparticles released from activated platelets expressing the DAMP HMGB1 were found in the blood of SSc patients.^462^ These microparticles lead to neutrophil activation and the generation of NETs, which are further degraded in the presence of BoxA (a competitive inhibitor of HMGB1), indicating the activation of platelet‐specific neutrophils through HMGB1.^462^ EC damage and platelet activation result in persistent vascular abnormalities, which are a key clinical feature of SSc, also known as scleroderma.^463^ Microparticles released from activated platelets in the blood of SSc patients (SSc‐microparticles) are enriched and express the DAMP HMGB1.^464^ In the presence of the competitive inhibitor BoxA for HMGB1, the interaction between microparticles and neutrophils, neutrophil autophagy and survival, as well as the generation of NETs, are weakened. Consistent with these findings, neutrophils in the blood of SSc patients undergo autophagy, and there is an abundance of NET byproducts. Thus, the results suggest an association between NETs and vascular abnormalities in SSc.^465^ Activated P‐selectin+ platelets and platelet‐derived HMGB1+ microparticles accumulate in the blood of SSc patients, but not in the blood of healthy controls.^466^ Additionally, HMGB1+ microparticles induce the production of NETs in the plasma of SSc patients.

### 6.16 Polymyositis/dermatomyositis and ILD

Polymyositis/dermatomyositis (PM/DM) is a group of idiopathic inflammatory myopathies primarily affecting skeletal muscles and characterized by nonsuppurative inflammatory infiltrates. It is often accompanied by ILD, with an incidence rate of 40–60%.^467^, ^468^, ^469^, ^470^, ^471^ Due to the high incidence and mortality rates of PM/DM‐related ILD, and the severity of the disease, there is a need to explore the pathogenesis of this refractory condition, making it a recent research focus.^472^ Recent studies have found the involvement of various immune‐inflammatory cells in the inflamed muscle tissues of PM/DM, including T cells, B cells, macrophages, dendritic cells, and neutrophils.^473^, ^474^ The phenotype and effector molecules of these immune cells can be altered in skeletal muscles, peripheral blood, and other organs, leading to organ involvement such as skin and lungs.^475^ A recent study showed that the formation of NETs is significantly increased in the plasma of DM patients and that the impaired clearance of formed NETs may play a role in DM and DM‐related ILD.^476^ Zhang et al.^477^ discovered that NETs induced EC injury in DM and associated ILD. Peng et al. studied patients with clinical amyopathic DM, classic DM, PM, and healthy controls and found that the formation of aberrant NETs may be involved in the pathogenesis of ILD in DM patients with anti‐MDA5 AAbs.^478^ Jiram et al. found that LDGs and NETs were significantly higher in patients with active disease, ulcers, calcinosis, and anti‐MDA5 antibodies, and were positively correlated with IL‐17A and IL‐18 levels. LDGs and NETs were higher in patients with calcinosis, increased antinuclear antibody titers, and positive anti‐PM/Scl75 tests. The proportion of total LDGs to NETs was higher in patients with calcinosis and skin ulcers. LL‐37 was higher in NETs derived from LDGs. The normal‐density neutrophil population was elevated in patients with active DM.^479^ Zhang et al. found that DM/PM patients had enhanced capacity to induce NETs compared with the control group, which was supported by increased levels of LL‐37 and cfDNA in DM/PM plasma. The degradation of NETs and DNase I activity were significantly reduced in DM/PM patients, and the reduction was positively correlated. Furthermore, DM/PM patients with ILD exhibited the lowest degradation of NETs in vitro, possibly due to decreased DNase I activity. Patients with anti‐Jo‐1 antibodies had significantly lower DNase I activity compared with patients without anti‐Jo‐1 antibodies. Steroid therapy appeared to improve DNase I activity. These findings suggest that impaired clearance of excessive NETs, particularly in patients with ILD, may be implicated in the pathogenesis of DM/PM and could be one of the factors contributing to the development and exacerbation of ILD.^480^ Recent studies have shown that NETs play a role in promoting fibroblast differentiation and function, thereby contributing to the development of pulmonary fibrosis in ILD associated with PM/DM.^481^ Further research has revealed that NETs can induce pulmonary fibrosis in PM/DM‐related ILD through the TLR9–miR‐7–Smad2 pathway.^482^, ^483^, ^484^, ^485^ Bhargavi et al. discovered a novel mechanism involving calcium crystal‐mediated neutrophil activation and cell death in the pathophysiology of chronic JDM. Using electron microscopy, they found the infiltration of neutrophils into affected muscle tissues and the ingestion of deposited calcium crystals. The uptake of crystals led to neutrophil activation and subsequent production of NETs that were independent of PI3K and NOX but dependent on PAD4. The formed NETs contained mitochondrial DNA and were confirmed in vitro and in vivo. Peripheral NET levels were associated with calcinosis, ICs, and IL‐8 levels in JDM patients. NET clearance rates were impaired in JDM patients and correlated with their autoimmune antibody spectrum, including melanoma differentiation‐associated protein 5 (MDA5) and reduced complement C4 levels. Targeting the NET pathway could reduce the frequency and severity of calcinosis and prevent the long‐term development of complications, including atherosclerosis.^486^

### 6.17 Systemic‐onset juvenile idiopathic arthritis

Systemic‐onset juvenile idiopathic arthritis (SoJIA) is one of the seven subtypes of JIA.^487^ SoJIA is a systemic autoimmune inflammatory disease, and current research has shown that impaired phagocytic function, including neutrophils, is a major pathogenic mechanism in SoJIA. Therefore, the activity of SoJIA may also be related to the activation of neutrophils.^488^, ^489^, ^490^, ^491^, ^492^ Recent studies have also suggested that NETs, which contain a large amount of histones known as extracellular histones, play a critical role in inflammation and are involved in the pathogenesis of many diseases.^493^, ^494^, ^495^ Hu et al.^496^ found that serum histone levels were positively correlated with disease activity in SoJIA. Serum histones may come from NETs released by activated neutrophils. Free iron can activate neutrophils to produce NETs, which then release histones. This histone positive feedback loop may be one of the mechanisms underlying the development of secondary SoJIA or macrophage activation syndrome (MAS). Histones may play an important role in the pathogenesis of SoJIA, and heparin can act on histones to prevent histone‐induced inflammation. Mor‐Vaknin et al. detected DEK in NETs spontaneously formed by synovial neutrophils in JIA patients and found that DEK targeting reagents reduced NET formation. DEK appears to be a key player in arthritis, and anti‐DEK reagents may be used for the treatment of JIA and other types of arthritis.^497^ Additionally, studies have shown that when SoJIA is active or when MAS occurs, macrophages increase the expression of CD163,^498^, ^499^ which may lead to elevated levels of free iron in the blood and subsequently activate neutrophils to release NETs, resulting in increased levels of histones in the serum. The elevated level of histones can further activate neutrophils to release NETs, thus inducing histone release again, forming a malignant positive feedback loop, increasing inflammation, and exacerbating disease progression. The level of extracellular histones may be a danger‐associated molecular pattern reflecting the degree of organ and tissue damage.^500^ Indeed, extracellular histones have been found to cause damage to the lungs, brain, liver, kidneys, and other organs.^501^, ^502^, ^503^, ^504^ Histones exacerbate tissue damage by killing other cells and activating TLR, NLRP3, and other inflammatory pathways. Therefore, histones are considered a potential therapeutic target for the treatment and control of inflammatory diseases.^505^, ^506^, ^507^ It has been found that heparin can neutralize the toxicity of histones in vitro and in vivo. In the treatment of sepsis, heparin has been proposed to act on histones to prevent histone‐induced inflammation and thus avoid further organ damage.^500^ This study also demonstrated that heparin effectively prevented the release of NETs by activated neutrophils stimulated by active SoJIA serum, histones, and FeCl3.

### 6.18 Spondyloarthritis

**FIGURE 9:**
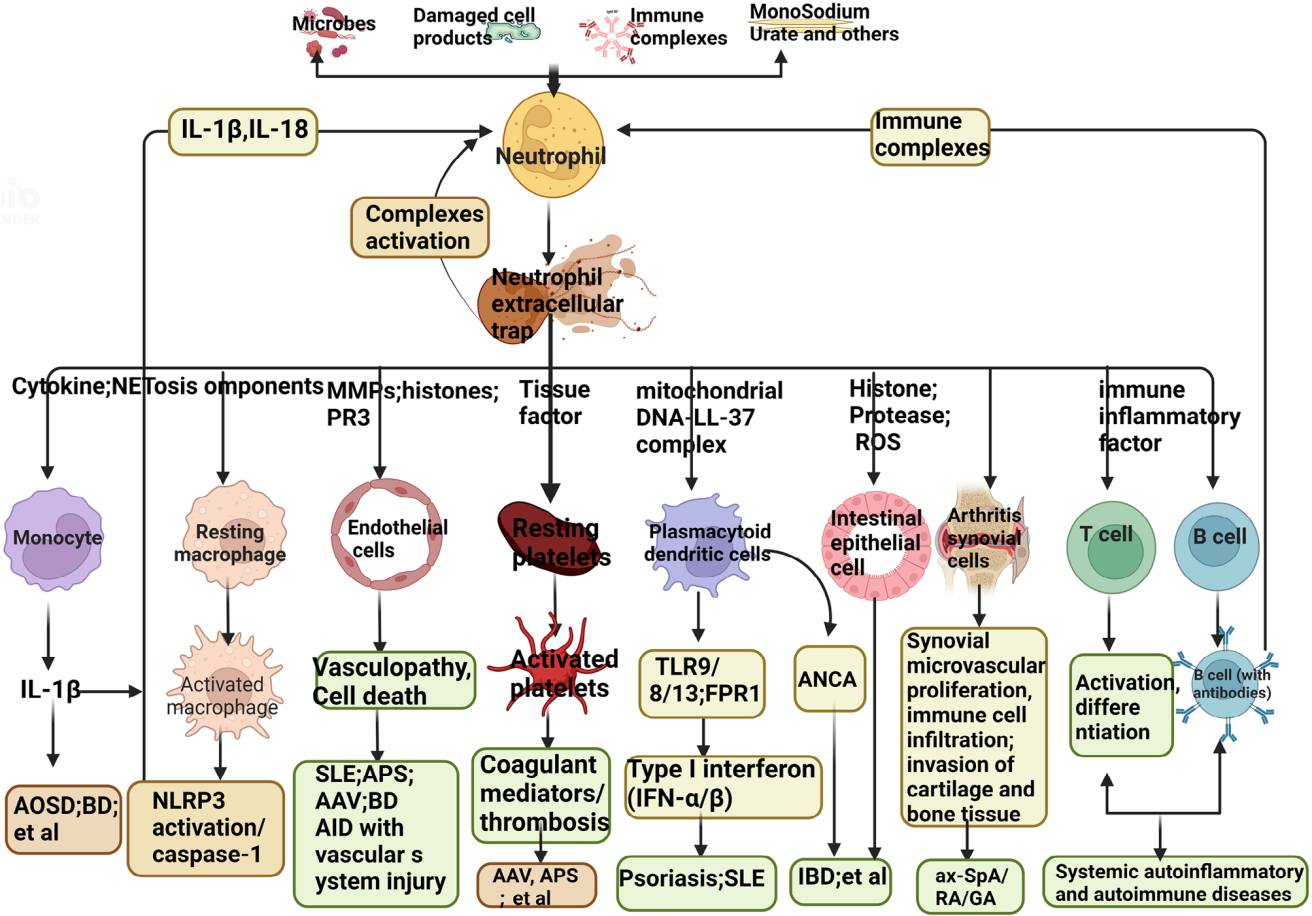
Mechanisms of neutrophil extracellular traps involved in spondyloarthritis. (Apoptotic neutrophils activate pDCs and mDCs to produce inflammatory cytokines and impair macrophage/monocyte phagocytosis, prolonging apoptosis and exacerbating damage. Enhanced NETosis in SLE, characterized by type I IFN production, aggravates vascular injury, suggesting neutrophil‐targeted therapies. NETs promote autoimmunity in RA and damage tissues via endothelial apoptosis and MMP activation, increasing inflammation and type I IFN. In IBD, neutrophils play dual roles, and targeting neutrophil‐microbiota interactions holds promise. Autophagy‐induced NETosis further regulates immunity, amplifying inflammation and autoimmune damage. Created by BioRender.)

Axial spondyloarthritis (axSpA) is an immune‐mediated inflammatory disease primarily affecting the axial skeleton and can also involve peripheral joints, entheses, and extra‐articular organs such as the eyes, skin, and intestines.^508^, ^509^, ^510^, ^511^, ^512^, ^513^ Studies have shown that inflammation contributes to bone erosion in axSpA‐associated ankylosis.^514^, ^515^ The major pathogenic mechanism of axSpA is believed to be impairment of the phagocytic function, including neutrophils. Therefore, the activity of axSpA may also be related to the activation of neutrophils.^492^ Recent studies have suggested that NETs may be released beyond activated neutrophils and contain abundant histones, which play a key role in inflammation and the development of a wide range of diseases.^493^, ^494^, ^495^ HU et al. found that serum histone levels were positively correlated with disease activity in axSpA.^496^ Serum histones may come from NETs released by activated neutrophils. Free iron can activate neutrophils to produce NETs, leading to the release of histones. This forms a positive feedback loop in which histones further promote the release of NETs, thereby exacerbating inflammation and disease progression. The level of circulating NETosis‐derived products (DNA, nucleosomes, and elastase) was found to be altered. Other studies have suggested that circulating NETosis‐derived products may serve as suitable biomarkers for distinguishing between r‐axSpA patients and healthy controls. Correlation studies have shown a relationship between circulating NETosis‐derived products and clinical inflammatory parameters. Furthermore, nucleosomes have the potential as a biomarker for distinguishing disease activity in r‐axSpA patients. IFX treatment was found to promote NETosis in axSpA patients and reduce disease activity. Mechanistic studies in vitro further revealed the correlation between reduced NET release and normalized enhanced inflammatory activity in mononuclear cells by IFX. In summary, neutrophils appear to play a novel role in the inflammatory process of AS, with IL‐17A‐modified NETs connecting MSC differentiation into osteoblasts. IL‐17A expression in neutrophils is regulated by IL‐1β. Blocking IL‐1β signaling on neutrophils or using DNase I to dismantle NETs disrupts osteogenesis driven by IL‐17A‐containing NETs. These findings shed light on a novel role for neutrophils in AS‐related inflammation, linking IL‐17A‐modified NETs to MSC differentiation into osteoblasts. Furthermore, IL‐1β triggers the expression of IL‐17A on NETs, providing an additional therapeutic target for AS^516^, ^517^, ^518^, ^519^, ^520^, ^521^ (Figure 9).

## 7 PROSEPCT

In recent years, growing evidence has highlighted the significant role of neutrophils in various inflammatory conditions. Neutrophils are central to the initiation and maintenance of autoimmune diseases through tissue damage, the creation of inflammatory environments, and the generation of new epitopes. Increasing recognition of their functional diversity suggests that defining specific neutrophil states in health and disease is a crucial area of research. Notably, NETs, key participants in both physiological defense mechanisms and pathological processes, underscore the critical role of neutrophils in human health and disease. While NETs provide essential antimicrobial defense, they also contribute to tissue damage and inflammation in numerous diseases. The interplay between NET‐mediated immune dysregulation, coagulation abnormalities, and tissue injury in autoimmune diseases is intricate, but many aspects of NET immunomodulatory properties remain poorly understood. Factors such as the local microenvironment, stimuli, and specific conditions of affected tissues and organs significantly influence NETs’ biological roles and functions.

Despite their importance, detailed research into neutrophil functions in autoimmune diseases faces numerous challenges. For example, neutrophils cannot be easily cultured in vitro, and their short half‐life in circulation limits the observation window for complex studies. Neutrophils are highly reactive to environmental stimuli, including pH, temperature, cytokines, and biomechanical properties. Moreover, NET formation occurs rapidly after neutrophil activation, within seconds to minutes, necessitating tailored experimental approaches to study these processes. Advances in single‐cell sequencing and spatial transcriptomics/proteomics hold promise for delineating neutrophil subpopulations within diseased tissues at unprecedented resolution. However, these technologies must address challenges in sampling, processing, and data analysis to accommodate neutrophils’ unique biological properties.^522^, ^523^

Current evidence suggests that many molecules released within NETs, such as double‐stranded DNA, histones, citrullinated peptides, MPO, and PRTN3, act as autoantigens targeted by the adaptive immune system in systemic autoimmune diseases. This implies a direct causal relationship between NETs and the induction or persistence of autoimmune conditions. NETs, as potential sources of autoantigens and immune cell activators, significantly contribute to autoimmunity and the breakdown of immune tolerance. Further research is needed to characterize NET structural components for designing novel autoimmune disease therapies. Understanding the impact of these components on innate and adaptive immune cells is essential for future investigations. Additionally, NET levels may serve as biomarkers for disease progression, treatment response, and patient relapse in autoimmune diseases, offering potential clinical utility. However, the roles of NETs in different tissue microenvironments and peripheral blood versus organ‐specific autoimmune conditions require further exploration.

Although extensive evidence supports neutrophil involvement in autoimmune and autoinflammatory diseases, few therapies specifically target neutrophil dysfunction. This limitation arises partly from the risk of increasing infection susceptibility through excessive immunosuppression. Ideal interventions should selectively target dysregulated neutrophil functions while preserving their essential antimicrobial roles. Novel therapies focusing on NET formation pathways rather than degrading already formed NETs may offer better outcomes. For instance, targeting factors initiating NET formation could prevent detrimental effects without compromising essential immune functions. Furthermore, developing dynamic in vivo strategies for NET modulation represents an exciting research avenue.

Future research should also prioritize defining neutrophil subpopulations, such as LDNs, which may play unique roles in autoimmune diseases and have minimal impact on general immunity when targeted. Technological advancements could enable the development of specific ligands targeting NETs or their components, providing new avenues for treating neutrophil‐driven inflammatory diseases. Progress in neutrophil biology is poised to revolutionize therapeutic strategies, offering substantial benefits to patients.

In summary, advancing our understanding of neutrophils’ roles in disease‐specific autoimmune conditions, including their contributions to disease initiation and secondary dysregulation, is imperative. Modern biotechnological innovations offer hope for developing targeted treatments, ultimately improving outcomes for patients with neutrophil‐associated disorders.

## AUTHOR CONTRIBUTIONS

Liuting Zeng, Wang Xiang, and Wei Xiao are responsible for the study concept and design. Liuting Zeng, Wang Xiang, Wei Xiao, Yang Wu, and Lingyun Sun are responsible for the data collection, data analysis, and interpretation. Liuting Zeng and Wang Xiang drafted the paper. Lingyun Sun supervised the study. All authors participated in the analysis and interpretation of data and approved the final paper.

## CONFLICT OF INTEREST STATEMENT

The authors declare no conflicts of interest.

## ETHICS STATEMENT

Not applicable.

## Data Availability

Not applicable.
